# Multidimensional role of adapalene in regulating cell death in multiple myeloma

**DOI:** 10.3389/fphar.2024.1415224

**Published:** 2024-08-08

**Authors:** Xinya Cao, Jie Xiang, Qi Zhang, Jinwen Liu, Dongming Zhou, Yong Xu, Peipei Xu, Bing Chen, Hua Bai

**Affiliations:** ^1^ Department of Hematology, Nanjing Drum Tower Hospital Clinical College of Nanjing University of Chinese Medicine, Nanjing, China; ^2^ Department of Pharmacology, School of Medicine, Yangzhou University, Yangzhou, China

**Keywords:** multiple myeloma, adapalene, bortezomib, CD138, programmed cell death

## Abstract

**Aims:**

Multiple myeloma (MM) remains a challenging condition to cure, with persistent drug resistance negating the benefits of treatment advancements. The unraveling complexities in programmed cell death (PCD), inclusive of apoptosis, autophagy, and ferroptosis, have highlighted novel therapeutic avenues. Our study focuses on deciphering how adapalene (ADA), a small molecule compound, accelerates the demise of MM cells via targeting their compensatory survival mechanisms.

**Methods:**

To assess the impact of ADA on MM, we employed flow cytometry and trypan blue exclusion assays to determine cell viabilities across MM cell lines and primary patient samples post-treatment. To delineate ADA’s therapeutic targets and mechanisms, we conducted RNA sequencing (RNA-seq), gene set enrichment analysis (GSEA), molecular docking, and molecular dynamics simulations. We further designed pre-clinical trials emphasizing MM, exploring the efficacy of ADA as a standalone and in combination with bortezomib (BTZ).

**Results:**

ADA elicited a dose-responsive induction of MM cell death. Building upon ADA’s anti-MM capabilities as a single agent, we proposed that ADA-BTZ co-treatment might amplify this lethality. Indeed, ADA and BTZ together greatly potentiated MM cell death. ADA proved beneficial in restoring BTZ susceptibility in BTZ-resistant relapsed or refractory MM (RRMM) patient cells. Molecular simulations highlighted ADA’s high affinity (−9.17 kcal/mol) for CD138, with MM-GBSA revealing a binding free energy of −27.39 kcal/mol. Detailed interaction analyses indicated hydrogen-bonding of ADA with CD138 at the Asp35 and Gln34 residues. Additionally, ADA emerged as a versatile instigator of both ferroptosis and apoptosis in MM cells. Furthermore, ADA disrupted activation of the nuclear factor kappa-light-chain-enhancer of activated B cells (NF-κB) pathway triggered by BTZ, fostering cell death in BTZ-resistant MM subsets.

**Conclusion:**

ADA demonstrates a comprehensive capability to orchestrate MM cell death, exerting pronounced anti-MM activity while disrupting NF-κB-related drug resistance. ADA sensitization of MM cells to BTZ unravels its potential as a novel therapeutic drug for MM management.

## Introduction

Multiple myeloma (MM), a complex hematologic malignancy, remains incurable with the therapies presently available. Initial treatment protocols involving proteasome inhibitors and immunomodulatory drugs have shown efficacy in triggering MM cell death. Nonetheless, the disease often reemerges, with the majority of patients relapsing due to the development of drug resistance (Chauhan and Anderson, 2003; Röllig et al., 2015). In the face of therapeutic interventions, MM cells can activate a range of compensatory mechanisms that evade cell death and facilitate tumor progression. The up-regulation of some transcription factors and mediators such as CD138, BCL-2, NF-κB, STAT3 and retinoic acid receptor (RAR), is known to thwart cell death in MM, thereby contributing to resistance and evasion of programmed cell death (PCD) pathways including ferroptosis, apoptosis, pyroptosis, and autophagic cell death (Wang et al., 2009; Kim et al., 2017; Guo et al., 2021). To overcome such resistance, one strategy involves the use of adjuvants specifically aimed at promoting MM cell mortality.

Retinoids have been identified as crucial mediators in PCD, impacting MM cells through the activation of RAR (Li et al., 2019). Among the three retinoic acid receptors (RARα, RARβ and RARγ), Zhan. et al. showed that RARα plays a significant role in regulating MM cell growth and disease progression by mediating the anti-MM effects of all-trans retinoic acid (ATRA) (Wang et al., 2009). Additionally, nuclear transcription factors RARβ and RARγ are implicated in the pharmacological activity of adapalene (ADA), a third-generation retinoid with noted anti-cancer effects (Milanese et al., 2011; Lee et al., 2022). ADA, a stable derivative of naphthoic acid, has been shown to modulate cell proliferation and differentiation through retinoid signaling pathways (Shroot and Michel, 1997). Besides its conventional applications, ADA can potentiate tumor cell suppression by influencing other PCD-related processes (Li et al., 2019; Wang et al., 2019; Nong et al., 2022). Currently marketed for the treatment of dermatological conditions, ADA’s repurposing for MM therapy epitomizes drug repositioning: the innovative application of established medications to new indications (Jourdan et al., 2020). Drug repositioning has the potential to streamline the drug development timeline and reduce associated costs, a critical consideration in light of prevailing healthcare challenges like the COVID-19 pandemic (Wang, 2021; Vaz et al., 2023).

Despite the therapeutic promise of ADA, its application in MM treatment remains to be reported. Therefore, this study aims to investigate the pharmaceutical effects and underlying molecular mechanisms of ADA, focusing on its capacity to modulate various types of cell death in MM.

## Methods

### Cell culture

Human H929 and LP-1 MM cell lines, along with primary MM cells were cultured in RPMI-1640 medium supplemented with 10% fetal bovine serum and 100 IU/mL of penicillin-streptomycin, with a CO_2_ incubator at 37°C. The H929 and LP-1 cell lines were kindly gifted by Dr. Chunyan Gu (Nanjing University of Chinese Medicine, China). Primary MM cells were sourced from patients newly diagnosed MM (NDMM) or with relapsed or refractory MM (RRMM), with sample collection approved by the ethics committee of Nanjing Drum Tower Hospital.

### Cell viability assay

MM cells were subjected to treatment with/without ADA (MedChemExpress, Shanghai, China; ADA was dissolved in dimethyl sulfoxide (DMSO)), both with and without bortezomib (BTZ) (MedChemExpress, Shanghai, China) for 24 h. Following treatment, a hemocytometer and the trypan blue exclusion assay (0.4% dye in phosphate-buffered saline, pH7.3) were employed to evaluate cell viability. The CCK-8 assay (Dojindo, Kumamoto, Japan) was used to quantify the viable cells, calculating viability as the ratio of live to total cells.

### Flow cytometry analysis

The detection of apoptosis was carried out using the Annexin V-FITC/PI apoptosis detection kit (Vazyme, Nanjing, China) according to the manufacturer’s instructions. Briefly, 5*10^5^ cells/well were treated with 40 μmol/L ADA, and at 24 and 48-h intervals, they were prepared for quantification via a BD flow cytometer (BD Biosciences, San Jose, United States) and subsequent analysis with FlowJo software. Primary bone marrow mononuclear cells (BM-MNCs) from the NDMM and RRMM patients were treated with ADA and/or BTZ overnight. They were then labeled with CD138-PE-conjugated antibody (Beckman Coulter Immunotech, Marseille, France) for 15 min before the percentages of CD138^+^ MM cells were obtained using a Beckman Coulter flow cytometer (Beckman Coulter, Fullerton, United States).

### RNA sequencing and GEO database analyses

H929 cells treated with either DMSO or ADA, were subject to RNA sequencing (RNA-seq) using the Illumina NovaSeq6000 (OE Biotech, Shanghai, China). Subsequent bioinformatics analyses were conducted via the OECloud platform. We used the GEO database to examine the expression and clinical relevance of SDC1 in MM patients, employing the datasets under the accession numbers GSE5900 (Zhan et al., 2007) and GSE2658 (Zhan et al., 2006).

### Western blot analysis

Whole cell lysates were resolved using 8%–12% SDS-PAGE, and transferred onto PVDF membranes (Millipore, Billerica, United States). Following blocking with 5% nonfat milk in Tris Saline with Tween, the membranes were incubated overnight with primary antibodies against PRAP, GAPDH, p65 (T40050, M20006, T55034, Abmart, Shanghai, China), FL-Caspase 3 (9,662, Cell Signaling Technology, Beverly, United States), cleaved-Caspase 3 (4ab080040, 4A Biotech, Beijing, China), BCL-2, GPX4, SLC7A11, CD138, IKKβ, IKBα (12789-1-AP, 67763-1-lg, 26864-1-AP, 10593-1-AP, 15649-1-AP, 10268-1-AP, Proteintech, Wuhan, China), Phospho-IKBα, and Phospho-p65 (AF300151, AF01236, Aifang, Hunan, China), followed by incubation in the corresponding secondary antibodies for 1 h at room temperature. Immunoreactive bands were visualized using an enhanced chemiluminescence kit (Sciben Biotech, Nanjing, China).

### Intracellular Fe^2+^ detection

A FerroOrange fluorescent probe (Dojindo, Kumamoto, Japan) was utilized according to the manufacturer’s protocol for the detection of intracellular Fe^2+^. In brief, 5*10^5^ cells/well were seeded in 6-well plates and treated with ADA with or without deferoxamine mesylate (DFOM). After 24 h, the H929 cells were rinsed and incubated with 250 μmol/L FerroOrange probe at 37°C for 30 min. Samples were then imaged by inverted fluorescence microscopy (Olympus, Tokyo, Japan).

### Molecular docking and MM-GBSA calculation

Since the two-dimensional structure of CD138 (syndecan-1) was not reported previously, the protein sequence modeling, homology detection and structure prediction were conducted by using HMM-HMM comparison provided by the Max-Planck Institute of Developmental Biology (http://toolkit.tuebingen.mpg.de/hhpred) with syndecan-4 (PDB: 1EJP; 61% sequence identity) as the closest structural homolog. Subsequently, molecular docking was conducted using Auto Dock Vina (Waterhouse et al., 2018). The grid box was generated to cover the entire receptor with the receptor center as the grid center. Gasteiger atomic partial charges were assigned for the investigated ligands. The docking results were analyzed and visualized using PyMOL (Rigsby and Parker, 2016). Binding free of ligand with protein using force-feld calculations was calculated using molecular mechanics-generalized born surface area (MM-GBSA) approach with a GB model (Chen et al., 2016).

### Molecular dynamics simulations

All molecular dynamics (MD) simulations were run only on the ligand and receptor using Gromacs 5.14 software. The peptide-membrane complexes were first solvated with SPC water molecules and positioned in the center of a cubic box. The solvated complexes were then subjected to energy minimization for 5,000 steps using a combination of steepest descent and conjugate gradient algorithms. The minimized systems were afterward smoothly heated from 0 to 300 K over 50 ps with a weak restraint of 10 kcal/mol/Å on the receptor. Further MD simulations were executed for 1,000 ps to equilibrate the investigated complexes. Eventually, MD simulations for each complex were conducted for 100 ns In MD simulations, the Particle Mesh Ewald (PME) method was applied for treating the long-range electrostatic interactions under periodic conditions with a direct space cutoff of 12 Å. In addition, the linear constraint solver (LINCS) algorithm was applied for covalent bond constraints. In order to maintain the temperature at 300 K, Langevin dynamics with collision frequency gamma_ln set to 1.0 was applied. The pressure was controlled using a Berendsen barostat with a relaxation time of 2 ps Bonds involving hydrogen atoms were also constrained via a SHAKE algorithm with a time step of 2 fs All MD simulations were carried out using a CPU version of gmx_mdrun tool, implemented in gromacs software. The pymol software was employed for 3D and 2D visualizations of the complex interactions.

### Statistical analysis

All statistical analyses were conducted using GraphPad Prism 6 (GraphPad Software, Inc., United States). To compare two groups, a two-tailed unpaired t-test was used. The Kaplan-Meier method was used for survival analyses. Drug Synergy was quantified according to the Chou-Talalay Method for non-constant drug combination using the CompuSyn software (Di Veroli et al., 2016). A combination index (CI) < 1 suggested synergistic effect. Differences were considered statistically significant if a *P* value was <0.05 (*), <0.01 (**), <0.001 (***) or <0.0001 (****).

## Results

### ADA exhibits promising cytotoxicity in MM cells

**FIGURE 1:**
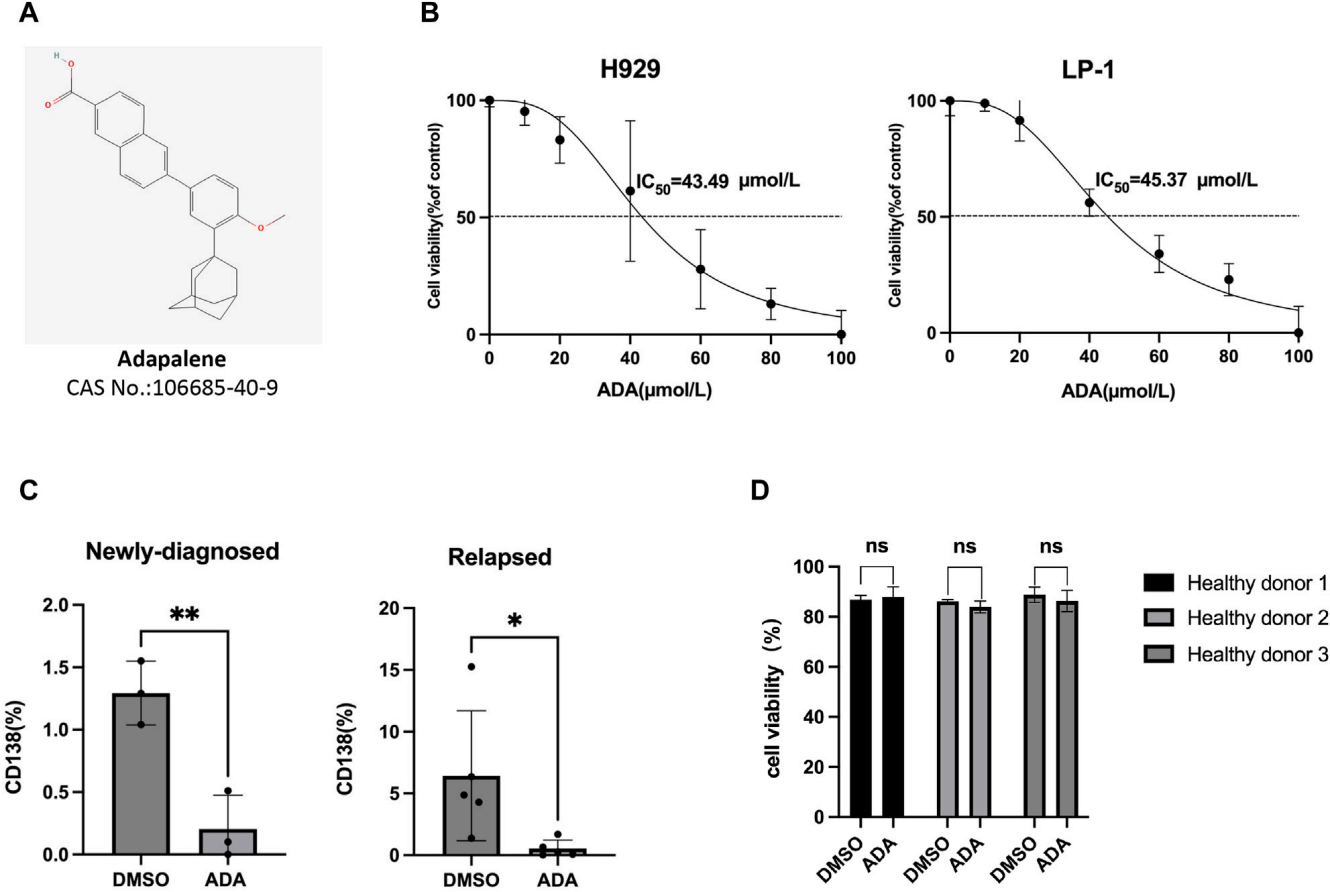
MM cell death induced by ADA. **(A)** The molecular structure of ADA. **(B)** Viability of MM cells treated with ADA by trypan blue exclusion assay. **(C)** ADA (40 μmol/L) treatment on primary BM-MNCs from NDMM and RRMM patients, with subsequent flow cytometric measurement of CD138^+^ cell percentages. **(D)** Primary BM-MNCs from healthy donors treated with ADA (40 μmol/L), and evaluated for cell viability by trypan blue. Statistical significance denoted as *P* < 0.05 (*), *P* < 0.01 (**).

The chemical structure of ADA {6-[3-(1-adamantyl)-4-methoxyphenyl]naphthalene-2-carboxylic acid, formula, C_28_H_28_O_3_, molecular weight, 412.52} is depicted in Figure 1A. We assessed ADA’s anti-MM potential by treating H929 and LP-1 cells with varying concentrations for 24 h. The results demonstrated that ADA induced MM cell death in a dose-dependent manner, with the average half-maximal inhibitory concentration (IC_50_) being 43.49 μmol/L for H929 cells and 45.37 μmol/L for LP-1 cells (Figure 1B). To extend our evaluation to primary clinical scenarios, BM-MNCs from NDMM and RRMM patients were treated with ADA. A consistent reduction in the percentage of primary CD138^+^ cells was observed in response to ADA treatment (40 μmol/L) (Figure 1C). Notably, ADA exhibited minimal cytotoxic effects on BM-MNCs from healthy donors, indicating selective therapeutic specificity toward MM cells without significant toxicity to normal cell counterparts (Figure 1D).

### Synergistic anti-MM effects of ADA and BTZ combination therapy

**FIGURE 2:**
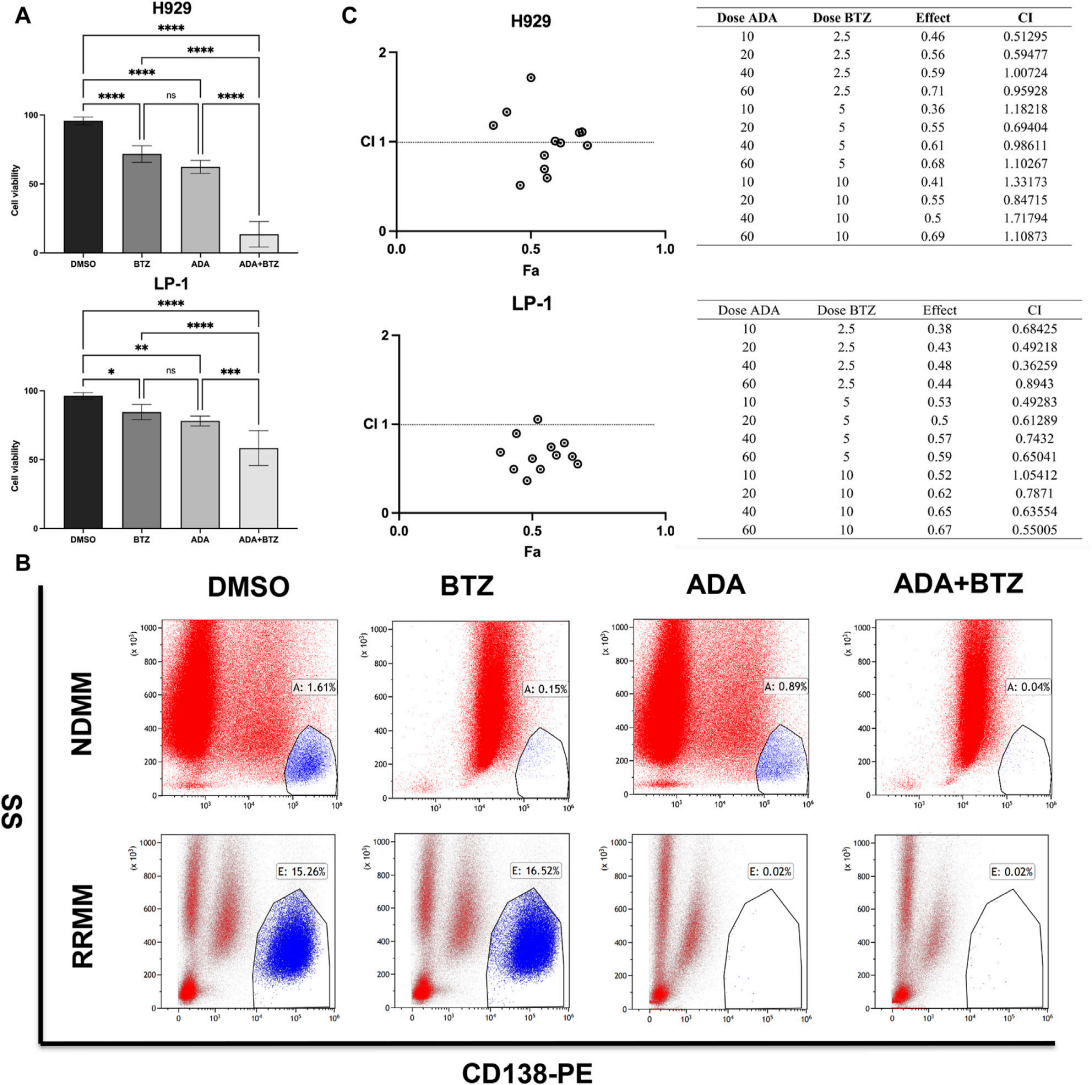
Synergistic anti-MM effect of ADA in combination with BTZ. **(A)** Viability of H929 and LP-1 cells by trypan blue exclusion assay after treatment with 40 μmol/L ADA, 5 nmol/L BTZ or the combination of ADA and BTZ. **(B)** Flow cytometry analysis of CD138^+^ cells in primary BM-MNCs from NDMM and RRMM patients treated with 40 μmol/L ADA, 5 nmol/L BTZ, and their combination. **(C)** Combination index of ADA and BTZ. H929 and LP-1 cells were treated 24 h across a range of concentrations of ADA, BTZ or ADA plus BTZ, and measured for cell viability using CCK-8 assay. CI values corresponding to the specified data points on the table were obtained using the CompuSyn software for non-constant drug ratio and plotted on the graph against fraction effect (Fa) to assess ADA-BTZ synergy. A CI < 1 indicates synergism. Statistical significance denoted as *P* < 0.05 (*), *P* < 0.01 (**), *P* < 0.001 (***), *P* < 0.0001 (****).

BTZ is a recognized first-line therapy for MM that effectively induces MM cell death (Cengiz Seval and Beksac, 2018). We hypothesized that combining ADA with BTZ could potentiate anti-MM effects, broadening clinical applications and enhancing therapeutic efficacy. To test this, H929 and LP-1 cells were treated with ADA and BTZ individually, or in combination, for 24 h, followed by evaluation of cell death. The combined treatment significantly elevated cell death percentages compared to single-agent treatments (Figure 2A). To further examine the anti-MM effects of this combination treatment on patient primary samples, BM-MNCs from NDMM and RRMM patients were treated ADA (40 μmol/L) and BTZ (5 nmol/L), individually and in tandem. Subsequent flow cytometry showed that ADA significantly inhibited the expansion of CD138^+^ MM cells even in the RRMM samples (3 cycles of BTZ-based regimes). The combination of ADA with BTZ further reduced the CD138^+^ cell population (Figure 2B). Given ADA’s impact on both NDMM and RRMM cells, we further asked whether it could synergize with BTZ. Drug synergy was quantified using the combination index (CI), with a CI value < 1 indicating a synergistic effect. The synergy assay revealed that ADA remarkably increased MM cell susceptibility to BTZ-induced cell death across various concentrations during a 24-h treatment period, yielding multiple CI values < 1 (Figure 2C). Collectively, these results confirm ADA’s significant cytotoxicity and ability to counteract BTZ resistance in MM.

### ADA is a multidimensional regulator of MM cell death

**FIGURE 3:**
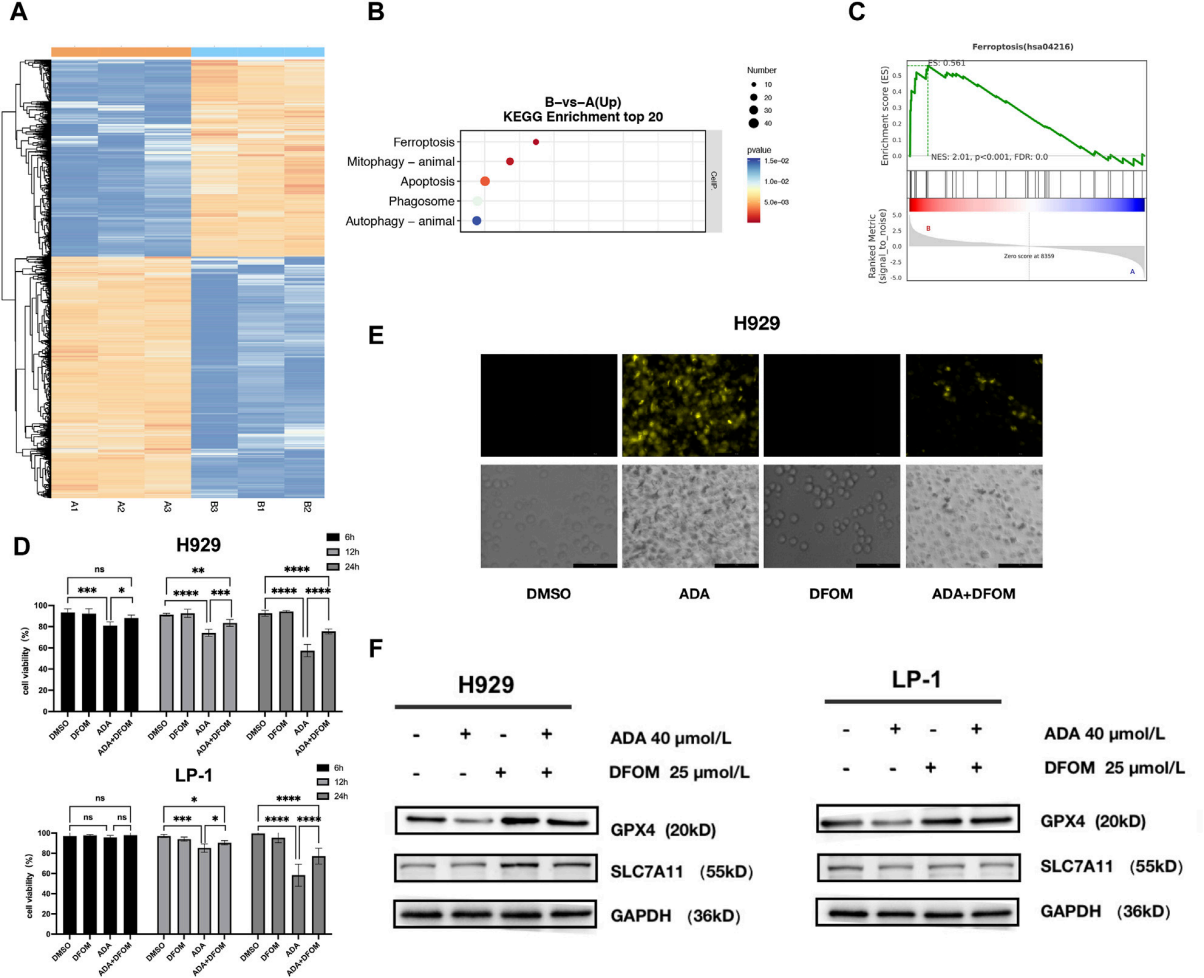
Multidimensional regulation of cell death by ADA. **(A)** Heatmap showing the differentially expressed genes (1924 upregulated and 2177 downregulated) in H929 cells treated with ADA. **(B)** KEGG pathway enrichment analyses conducted on upregulated DEGs. **(C)** GSEA analysis was performed to analyze pathways and function annotation. **(D)** The effects of ADA on the viability of MM cells in the presence or absence of DFOM. **(E)** Quantification of the level of Fe^2+^ in MM cells by FerroOrange probe. **(F)** The effects of ADA on the protein level of GPX4 and SLC7A11 were determined in the presence or absence of DFOM. Statistical significance denoted as *P* < 0.05 (*), *P* < 0.01 (**), *P* < 0.001 (***), *P* < 0.0001 (****).

To investigate the anti-MM mechanism of ADA, we examined the global transcriptional changes by RNA-seq data from H929 cells treated with ADA compared to the control (DMSO). The heatmap showed the differentially expressed genes (DEGs) in H929 cells treated with ADA versus DMSO (Figure 3A). There were 4101 DEGs (1924 upregulated and 2177 downregulated) in H929 cells (FC > 1, *P* < 0.05, edgeR). Among these DEGs, Kyoto Encyclopedia of Genes and Genomes (KEGG) enrichment analyses revealed that upregulated DEGs were mainly enriched in multiple cell death pathways, such as ferroptosis, mitophagy, apoptosis, phagosome, and autophagy pathways (Figure 3B), aligning with previous observations that ADA significantly propels MM cell death.

GSEA results confirmed the significant enrichment of DEGs in the ferroptosis pathway (Figure 3C). To validate ADA-induced ferroptosis, we employed the specific inhibitor, DFOM. Co-treatment with DFOM alleviated ADA’s anti-MM effects (Figure 3D), supporting the hypothesis. In line with this, intracellular Fe^2+^ levels, a hallmark of ferroptosis, increased following ADA treatment, a rise mitigated by DFOM (Figure 3E). Consistent with these findings, the protein levels of key ferroptosis markers, GPX4 and SCL7A11, decreased after ADA treatment, and again DFOM reversed this effect (Figure 3F), thus corroborating the induction of ferroptosis by ADA in MM cells.

**FIGURE 4:**
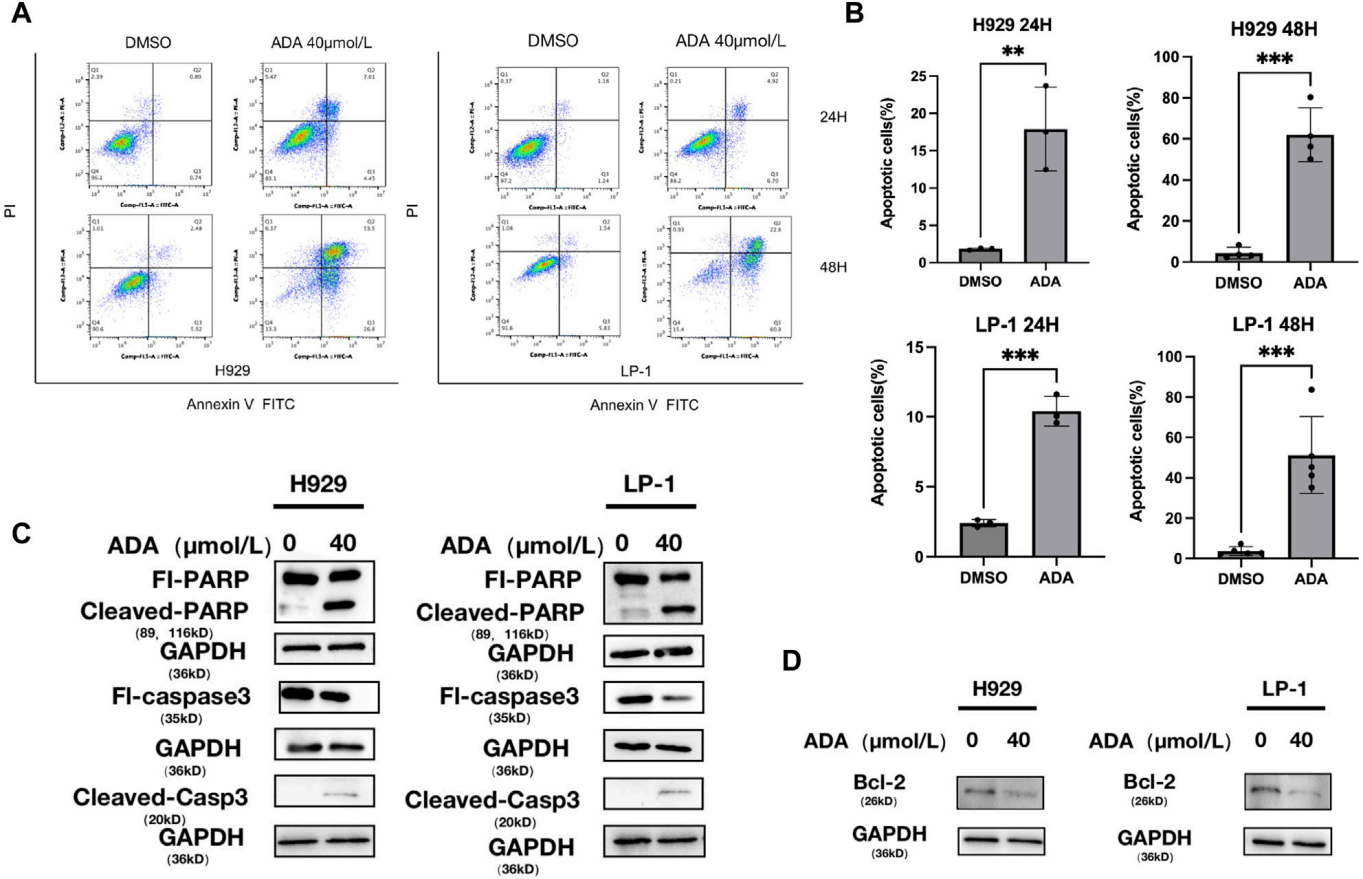
ADA induced the apoptosis of MM cells. **(A,B)** H929 and LP-1 cells were treated with ADA for 24 h or 48 h, followed by analysis for apoptosis with Annexin V/PI double staining. **(C,D)** Western blot was utilized to detect the level of apoptosis related proteins after ADA treatment. H929 and LP-1 cells were treated with ADA for 24 h; protein lysates were subjected to immunoblotting with the indicated antibodies.

In KEGG analysis, the apoptosis pathway came into focus among the upregulated DEGs, indicating its crucial role in ADA-provoked MM cell death. Diving deeper, we assessed apoptosis using Annexin V-FITC/PI staining (Figure 4A). ADA elevated apoptosis rates markedly compared to the control group (Figure 4B), and promoted the protein expression levels of PARP and cleaved-Caspase 3, signifying the onset of apoptosis (Figure 4C). Additional, ADA significantly downregulated BCL-2 expression in MM cells (Figure 4D), confirming its potential to trigger apoptosis within this cellular context.

### ADA’s potent CD138 inhibitory activity against MM

**FIGURE 5:**
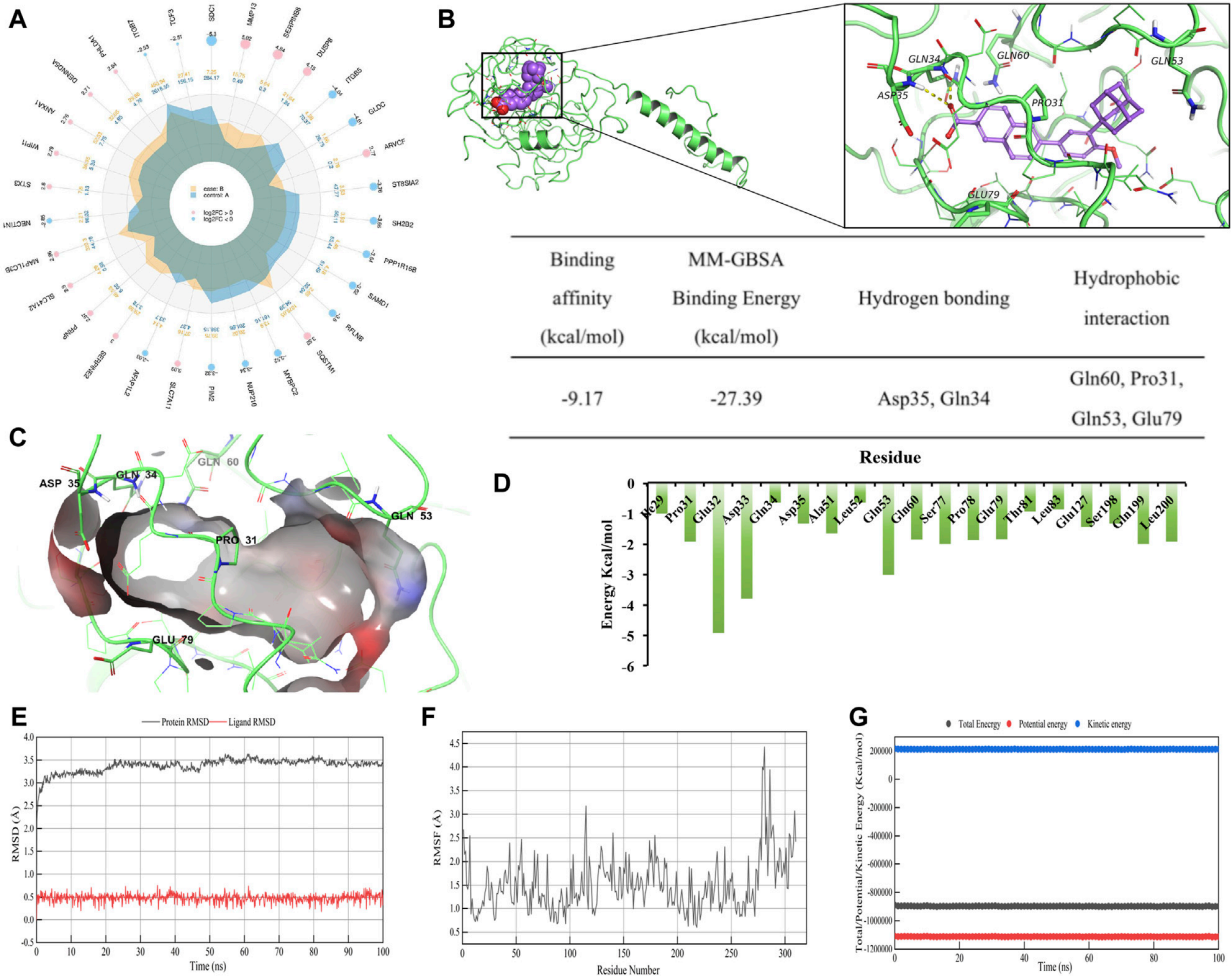
ADA is a CD138 inhibitor. **(A)** A radar plot showing the extent of fold changes among DEGs when comparing DMSO-treated and ADA-treated cells. **(B)** Computer-generated models of CD138’s active sites targeted by ADA. The residues of ligand bind domain (LBD) proteins representing hydrogen bonds are depicted and annotated as yellow dotted lines. The binding affinity, MM-GBSA binding energy (kcal/mol) and detailed intermolecular binding interactions of CD138 with ADA in molecular simulation regarding main hydrogen bonds and hydrophobic interactions. **(C)** 3D diagram of interaction between CD138 and ADA showed the major binding sites and bonding forces. **(D)** The energy contributions of each residue in CD138-ADA complex. **(E)** RMSD and **(F)** RMSF plot of ADA complexed with CD138 during 100 ns **(G)** Total, kinetic and potential energy variations in the MDs.

**FIGURE 6:**
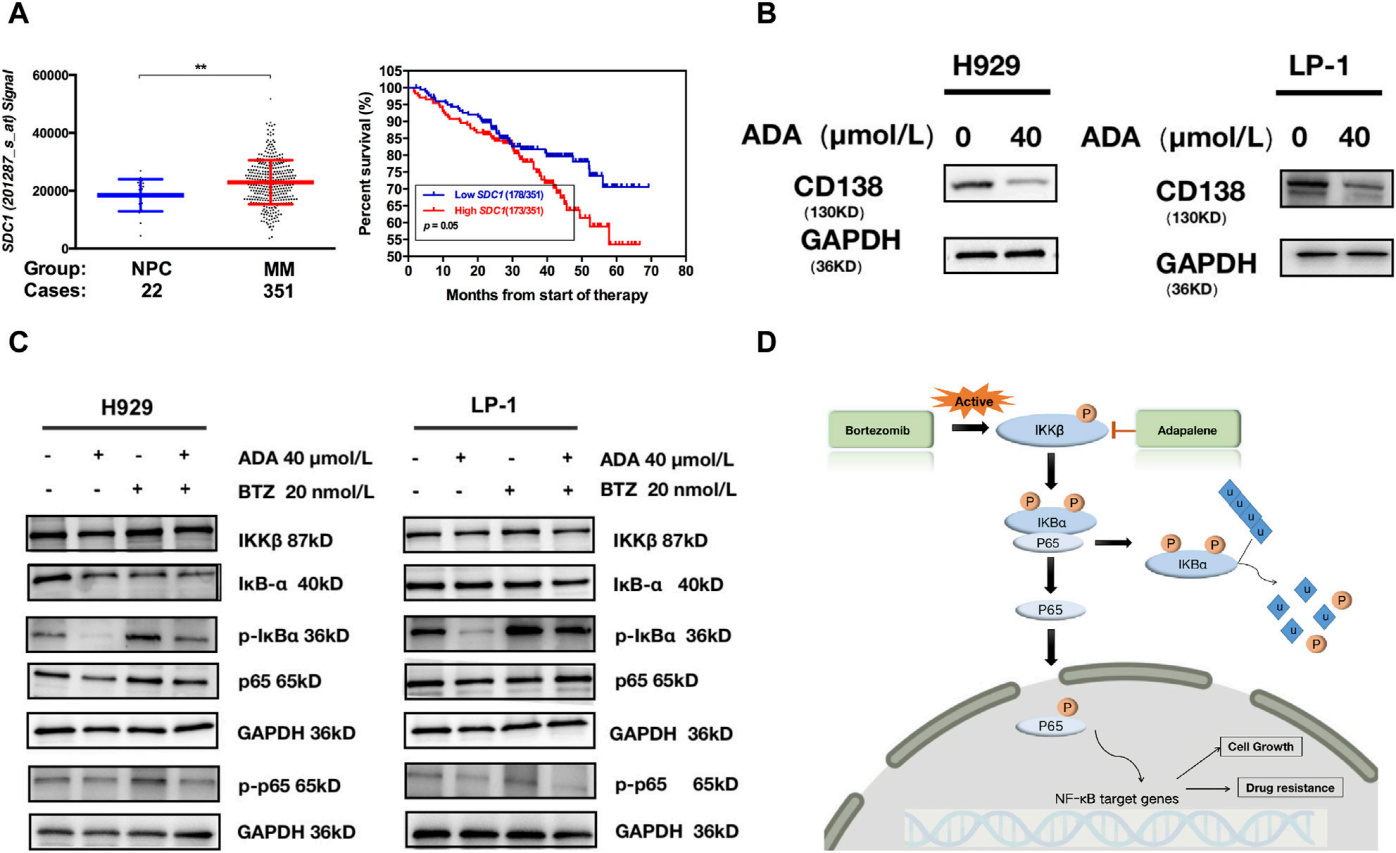
ADA inhibited CD138 and activation of the NF-κB pathway. **(A)** A scatter plot contrasting CD138 expression in normal plasma cell and MM subgroups; Kaplan-Meier analyses of overall survival revealed that high CD138 expression conferred inferior clinical outcomes in GSE2658 dataset. **(B)** Western blot was utilized to detect the level of CD138 after ADA treatment. H929 and LP-1 cells were treated with ADA for 24 h; protein lysates were subjected to immunoblotting with the indicated antibodies. **(C)** H929 and LP-1 cells were treated by single or both drugs, followed by Western blot to determine the expressions of the indicated proteins. **(D)** Illustrated depiction of ADA’s mechanistic effects on the NF-κB signaling cascade within MM cells.

One standout DEG was SDC1 (syndecan1, CD138), which showed a pronounced decreased (Figure 5A). SDC1 is inherently expressed at high levels on MM cell surfaces and is known to be shed into the tumor microenvironment, a feature linked to MM pathogenesis (Khotskaya et al., 2009). To evaluate the biological outcomes of elevated SDC1 expression in MM patients, we grouped all MM patients into two groups based on SDC1 expression and found that patients featuring lower SDC1 levels had improved overall survival outcomes compared to those with higher SDC1 levels (Figure 6A).

To further investigate whether there is a direct binding site and characterize binding between ADA and CD138, we performed 3D modeling of protein molecules based on homologous templates according to the protein sequence. The structure of CD138 (Uniport ID: P18827) was modeled using templates 1EJP (similarity: 61%). The docking model predicted that ADA has an affinity binding energy binding of −9.17 kcal/mol to CD138. Furthermore, MM-GBSA was utilized to predict the binding free energies of compound to the targeted protein. The estimated binding free energies (ΔG) from MM-GBSA calculations identified that ADA exhibits low glide energy of −27.39 kcal/mol against CD138. More specifically, ADA formed hydrogen bonds with CD138 upon Asp35 and Gln34 residues. Besides, there were four hydrophobic contacts contributed to the ADA-CD138 binding, namely, Gln60, Pro31, Gln53 and Glu79 (Figures 5B, C).

To explore the stability and dynamic interactions of protein-ligand complex, MDs were carried out to verify the results of the docking simulation and analyze the molecular movements of CD138 upon ADA binding. The docking complex was subjected to a 100 ns simulation, during which the temperature, kinetic and potential energies remained stable (Figures 5E–G). Figure 5E indicated that the RMSD of AS fluctuated between 0.30 and 0.80 Å, while the RMSD of the protein fluctuated between 3.00 and 3.50 Å. The RMSD value fluctuated less during the MD simulations, suggesting that the docking poses were reliable. From Figure 5F, we can see the qualitative residue-wise thermal fluctuations of dynamic models. The lower RMSF value is attributable to a significant number of H-bonding contacts and more non-bonding hydrophobic interactions. To identify the key amino acid residues involved in CD138 protein binding, the energetic decomposition of amino acid residues was performed. Figure 5D displayed the amino acid residues with energy contribution values below −1 kcal/mol, indicating that residues number 29–35, 51–53, 60, 77–83, 127, 198–200 of CD138 are the primary contributors to binding free energy, which are consistent with the docking result of key residues upon CD138-ADA binding complex. Complementing this, Western blot analysis demonstrated that ADA suppressed CD138 expression in both H929 and LP-1 cell lines (Figure 6B), establishing its robust anti-MM activity by inhibiting CD138.

### ADA inhibits activation of the NF-κB pathway

BTZ is a cornerstone in the management of MM, having markedly improved outcomes for NDMM patients (Cengiz Seval and Beksac, 2018). However, the inevitable emergence of drug resistance following prolonged BTZ exposure often results in relapse among RRMM patients (Qin et al., 2017). ADA monotherapy or in combination with BTZ successfully inhibited the growth of CD138^+^ MM cells in RRMM patients who had experienced three cycles of BTZ-based regimes, evidencing a markedly resistance to BTZ that ADA could counteract (Figure 2B). A well-recognized molecular mechanism involved in BTZ resistance is the activation of the canonical NF-κB pathway in MM cells (Hideshima et al., 2009), with increased NF-κB activity seen in MM patients who are refractory to BTZ treatment (Markovina et al., 2008). Exemplified in Figure 6C, BTZ administration initiates the NF-κB pathway cascade in both H929 and LP-1 cells. The addition of ADA significantly suppressed the activation of NF-κB induced by BTZ, underscoring the synergistic interaction between ADA and BTZ. As shown in Figure 6D, BTZ activates IKKβ, which subsequently phosphorylates IKBα. After proteasome degradation of IKBα, p65 moves to the nucleus to exert its functions. ADA blocks the phosphorylation of IKKβ, and inhibits the IKKβ-IKBα-p65 axis, which induces cell death in BTZ-resistant MM cells.

## Discussion

The therapeutic landscape of MM has experienced a remarkable shift with the integration of proteasome inhibitors, immunomodulatory drugs, and monoclonal antibodies into treatment regimens. These advancements have indeed improved outcomes but MM continues to challenge clinicians and scientist with its incurability (Chauhan and Anderson, 2003; Röllig et al., 2015). The evolution of our understanding regarding MM’s molecular intricacies suggests that deciphering these mechanisms might yield effective approaches to optimize future clinical outcomes. PCD pathways are increasingly recognized for their integral roles in MM pathogenesis; thus, they represent auspicious therapeutic targets (Taetle et al., 1996; Chen et al., 2018; Han et al., 2022).

The therapeutic application of retinoids in oncology is predicated on their activation of RARs, which are crucial in mediating their therapeutic effects (Bushue and Wan, 2010). Among the retinoic acid receptors (RARα, RARβ and RARγ), the co-administration of ATRA with arsenic trioxide represents a breakthrough front-line treatment for acute promyelocytic leukemia, acting via modulation of RARα activity, offering new avenues for effectively managing the disease (Ma et al., 2016). ADA, also known as differin and CD271, is recognized as a third-generation retinoid that received approval from the Food and Drug Administration (FDA) in 1996 for the treatment of acne vulgaris. ADA exerts its retinoid activity through RARβ and RARγ (Milanese et al., 2011; Lee et al., 2022). Its established properties include immunomodulation, anti-inflammatory actions, anti-proliferation, anti-bacterial effects, comedolytic action and neuroprotection (Rusu et al., 2020; Boulos et al., 2023). Multiple studies have highlighted ADA’s compelling anti-cancer properties (Ocker et al., 2003). ADA has been shown to induce cell death in hepatoma cells by regulating the ratio of BAX and BCL-2 (Ocker et al., 2004), to impede proliferation in ovarian cancer via GOT1 inhibition (Wang et al., 2019), and to exert pronounced anti-cancer activity in prostate cancer cells by inducing DNA damage, cell cycle arrest and apoptosis (Nong et al., 2022). Such evidence underscores the potential of repurposing ADA for cancer treatment.

Ferroptosis is a relatively recent discovery in the realm of PCD, rising as a fresh contender in cancer therapy (Kajarabille and Latunde-Dada, 2019). The efficacy of ferroptosis inducers in eliminating cancer cells, particularly those exhibiting drug resistance or mesenchymal and dedifferentiated traits, has been highlighted (Zhang et al., 2022). Apigenin, fingolimod, extracts from fumaria officinalis and thymus vulgaris, have been found to potentially exert anti-MM effects through ferroptosis induction (Zhong et al., 2020; Adham et al., 2021a; Adham et al., 2021b). Moreover, synergistic enhancement of anti-MM efficacy has been observed when ferroptosis inducers are combined with established frontline drugs, signaling new opportunities to improve patient outcomes (Chen et al., 2021; Li et al., 2022).

As noted previously, certain cancer therapy modalities can induce cell apoptosis to inhibit the proliferation of malignant cells (Evan and Vousden, 2001). An increased occurrence of apoptosis denotes a favorable tumor response to treatment (Dwivedi et al., 2020). In addition, genetic aberrations in cancer cells can result in the downregulation of pro-apoptotic genes (e.g., p53, BAX, PTEN) or the upregulation of genes related to cell survival (e.g., PI3K, BCL-2) (Huang et al., 2022). Through ADA treatment, which curtails survival-promoting genes like BCL-2 and activates pro-apoptosis mediators (PARP and cleaved-Caspase 3), effective induction of apoptosis in MM cells can be achieved.

CD138 is characterized by its elevated expression on mature plasma cells, making it a diagnostic target for MM (Yoo et al., 2015; Vasuthasawat et al., 2016). To develop novel targeted therapies for MM, a crucial strategy involves specifically targeting CD138; currently, no specific CD138 inhibitors are clinically approved. The identification of ADA as a CD138 inhibitor in our study represents an encouraging breakthrough, particularly as ADA has already been approved by the FDA and has a well-defined safety profile, as well as thoroughly explored pharmacodynamics and pharmacokinetics (Tolaymat et al., 2024).

NF-κB, a multifunctional transcription factor, plays pivotal roles in MM progression. Elevated NF-κB expression and its nuclear translocation are hallmarks of resistance to BTZ therapy (Markovina et al., 2008; Huynh et al., 2018). The degradation of NF-κB can be induced by stimulating the inhibitor of κB, resulting in the downregulation of anti-apoptotic genes (Mortezaee et al., 2019). Our results demonstrate ADA’s capacity to attenuate the NF-κB pathway activated by BTZ.

Our study has illuminated the therapeutic potential of ADA in inducing MM cell death, as evidenced by MM cell lines and primary patient-derived cells. Importantly, ADA was found to potentiate the effects of BTZ, suggesting a synergistic benefit when used in combination treatments. Mechanistically, ADA emerged as a composite regulator of cell death mechanisms in MM, promoting both ferroptosis and apoptosis while markedly inhibiting CD138 expression in MM cells. Additionally, ADA exhibited an inhibitory influence on NF-κB signaling cascade, a pathway critical for MM cell proliferation and drug resistance. Our investigation not only delineates the roles and molecular underpinnings of ADA in MM but also posits its significance as a promising therapeutic strategy. Building on these insights, our findings furnish a deeper understanding that could amplify the clinical effectiveness of MM treatments in the near future.

## Conclusion

The study emphasizes ADA’s valuable prospects as a versatile agent in the regulation of MM cell death. ADA demonstrates potent cytotoxicity by orchestrating a convergence of cell death mechanisms, including the induction of ferroptosis and apoptosis, the inhibition of CD138 expression, and the blockade of NF-κB pathway. The identification of ADA’s capacity to act as cell death regulator could substantially reduce the expenses associated with the development of innovative anti-MM drugs. Moreover, the insights gained on ADA’s mechanisms of action hold promise for refining ADA-based treatment regimes, which could lead to enhanced, targeted therapy strategies for both NDMM and RRMM.

## Data Availability

The original contributions presented in the study are publicly available. This data can be found here: https://www.ncbi.nlm.nih.gov/sra/PRJNA1034277.
