# Supplementary material for: Multidimensional role of adapalene in regulating cell death in multiple myeloma
Source: Front Pharmacol. 2024 Aug 8;15:1415224. doi: 10.3389/fphar.2024.1415224 (PMC11338798; doi:10.3389/fphar.2024.1415224)
Supplement: Supplementary file 1 [file DataSheet2.PDF]

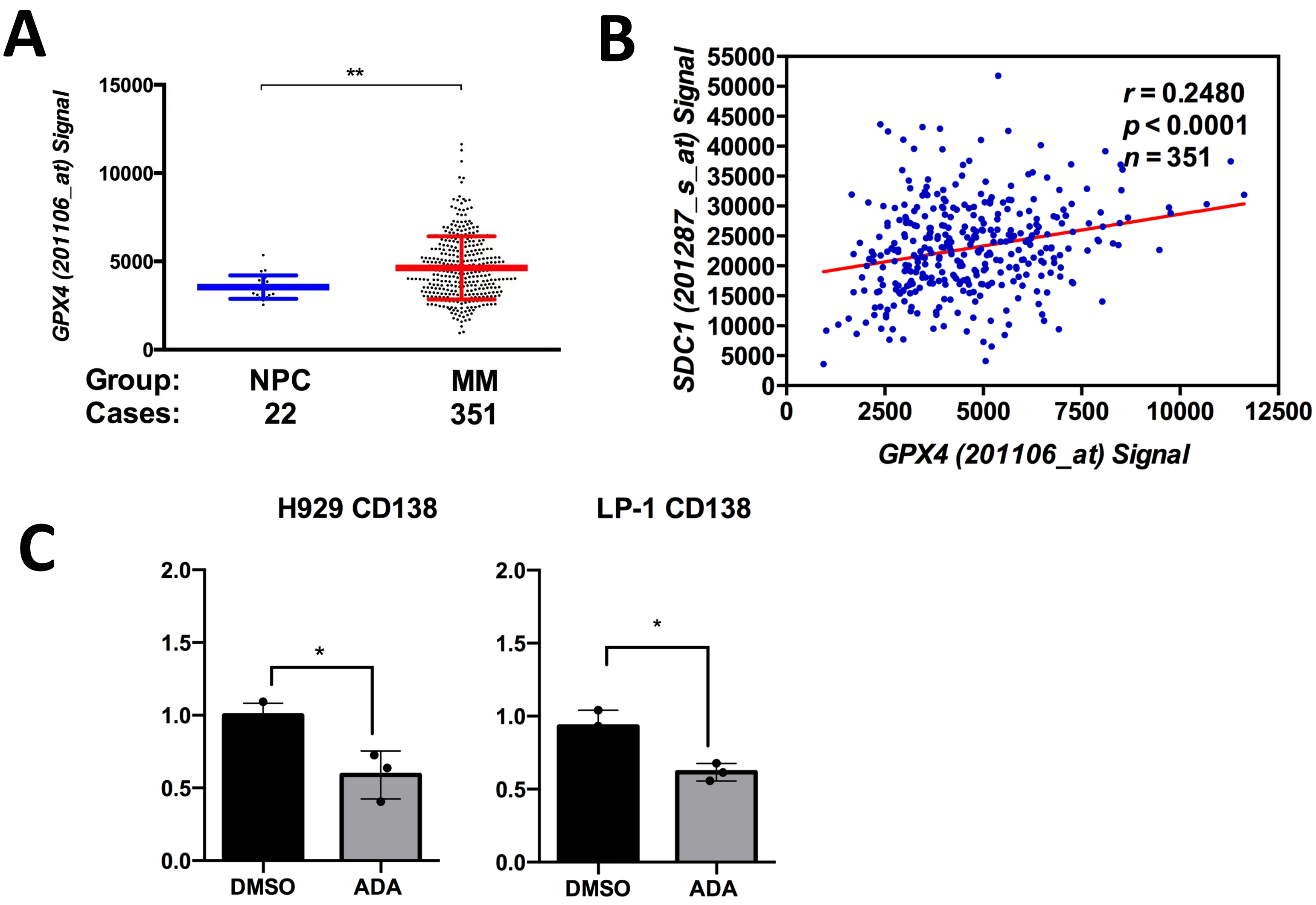

(A) The scatter plot shows GPX4 expression in Normal plasma cell and MM subgroups;

(B) A scatter plots demonstrating positive correlation of GPX4 and CD138 expression in MM patients.

(C) The blots for CD138 were semi-quantified.
