# Supplementary material for: Multidimensional role of adapalene in regulating cell death in multiple myeloma
Source: Front Pharmacol. 2024 Aug 8;15:1415224. doi: 10.3389/fphar.2024.1415224 (PMC11338798; doi:10.3389/fphar.2024.1415224)

Figure 3F

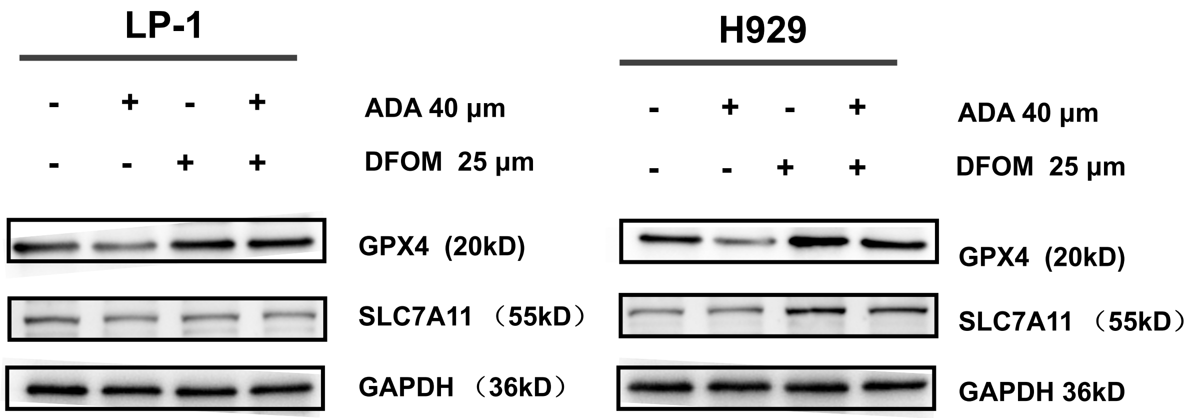

LP-1
H929
- + - +
ADA 40 μm
- - + +
DFOM 25 μm
GPX4 (20kD)
SLC7A11 （55kD）
GAPDH （36kD）
GAPDH 36kD

GPX4

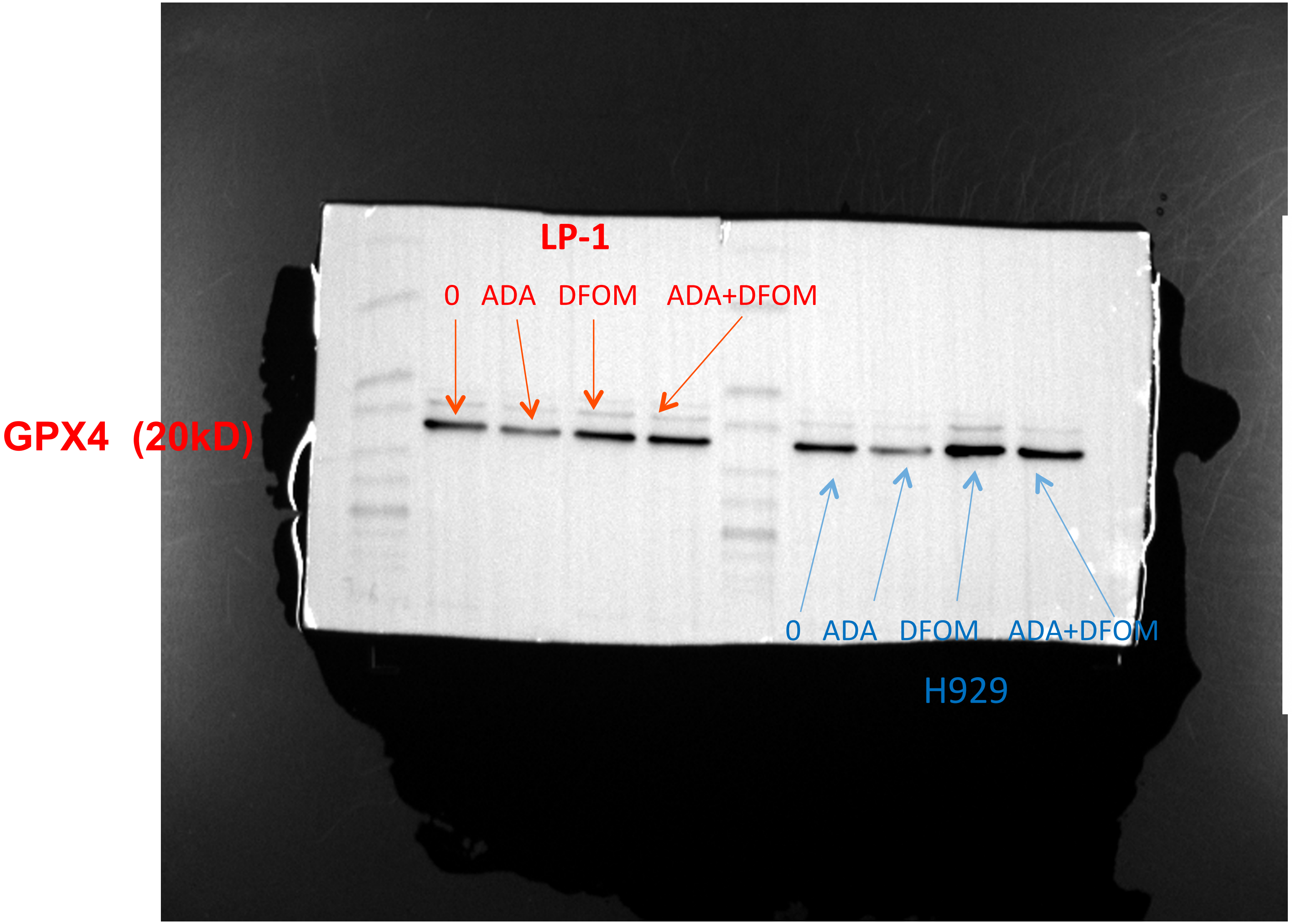

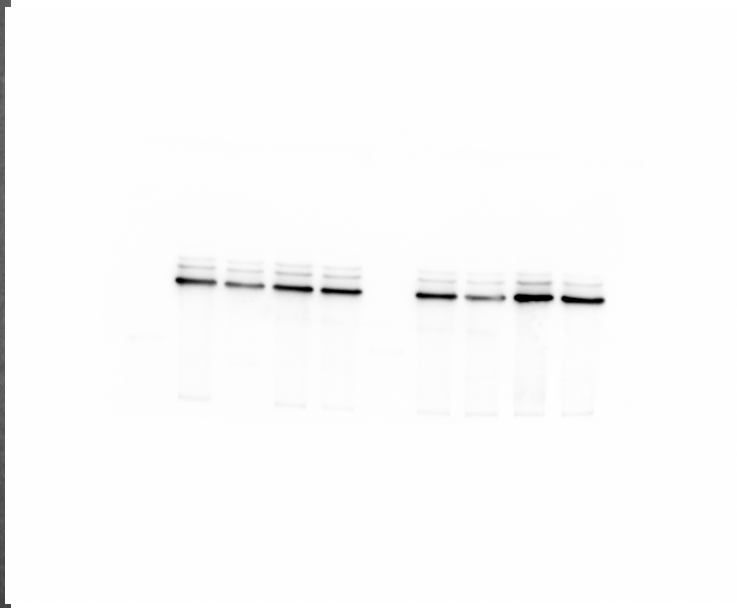

SLC7A11

SLC7A11 （55kD）

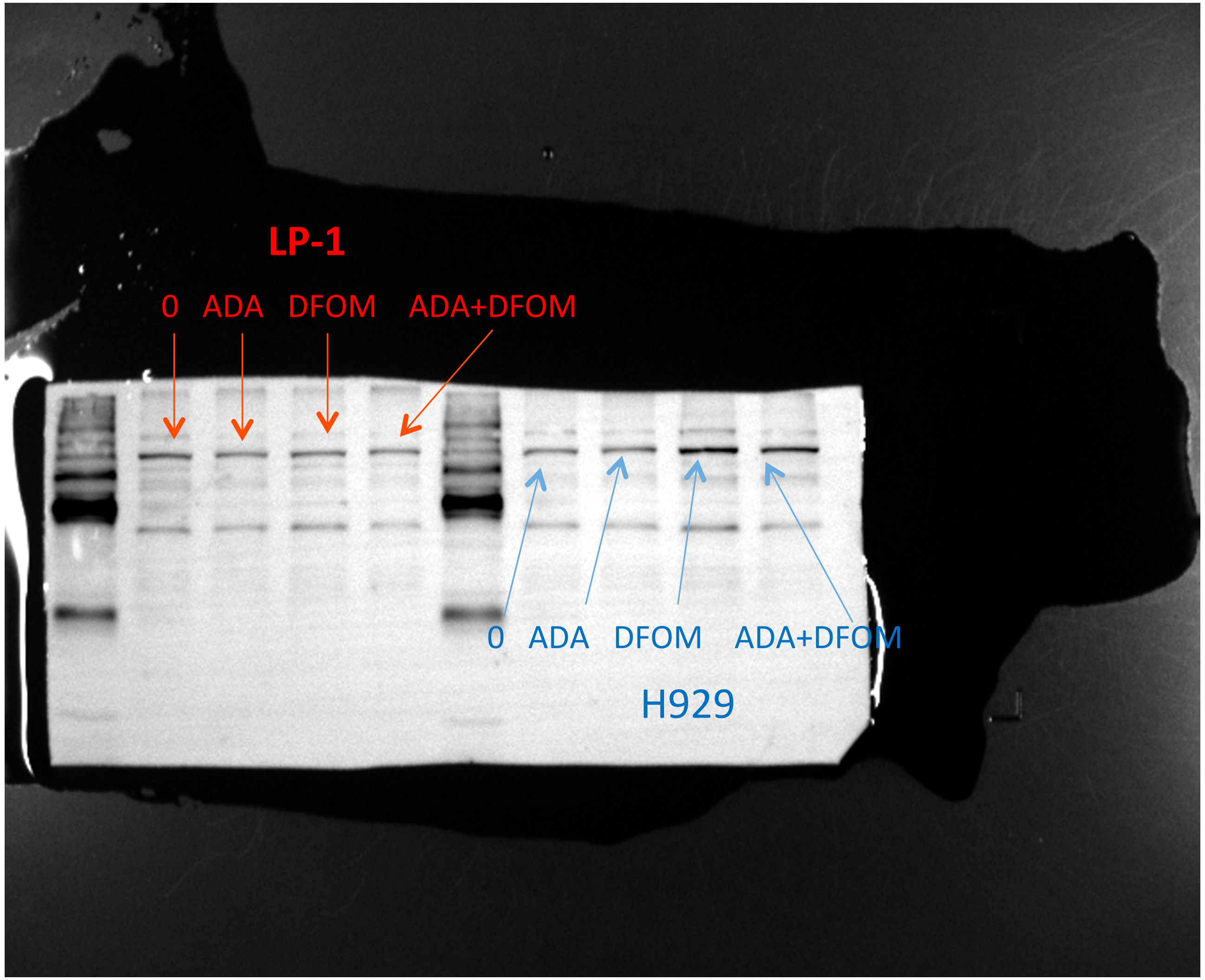

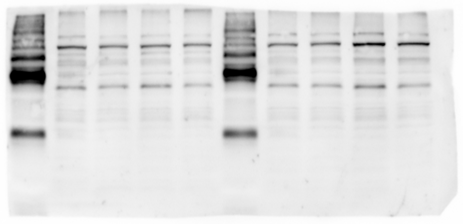

GAPDH

GAPDH（36kD）

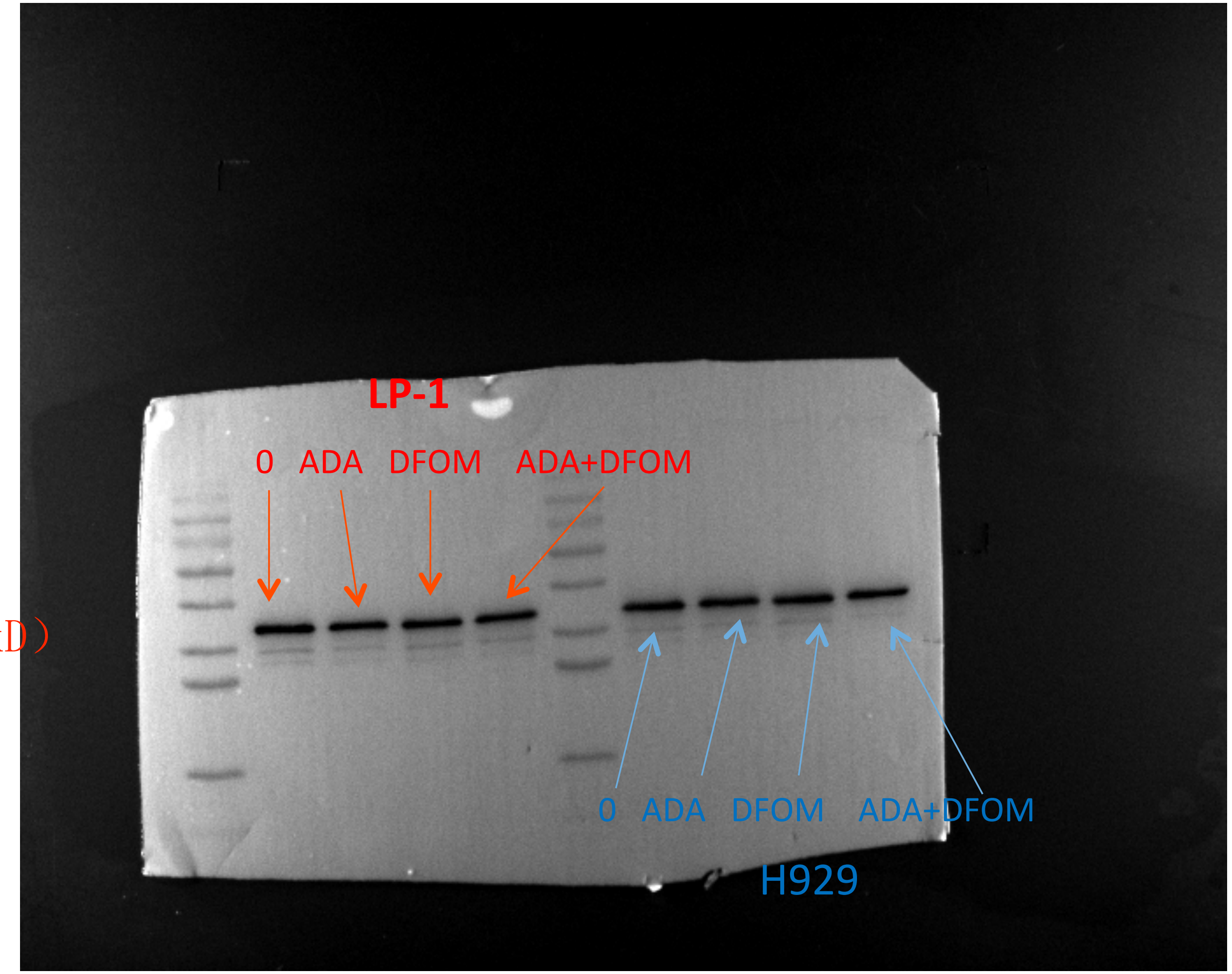

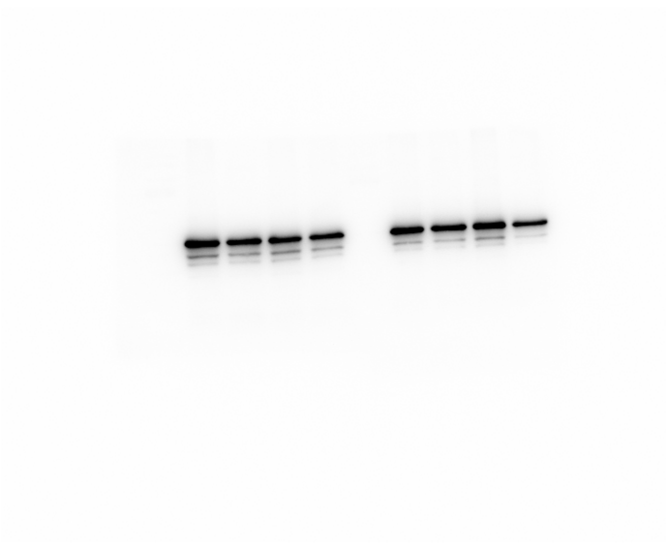

Figure 4C

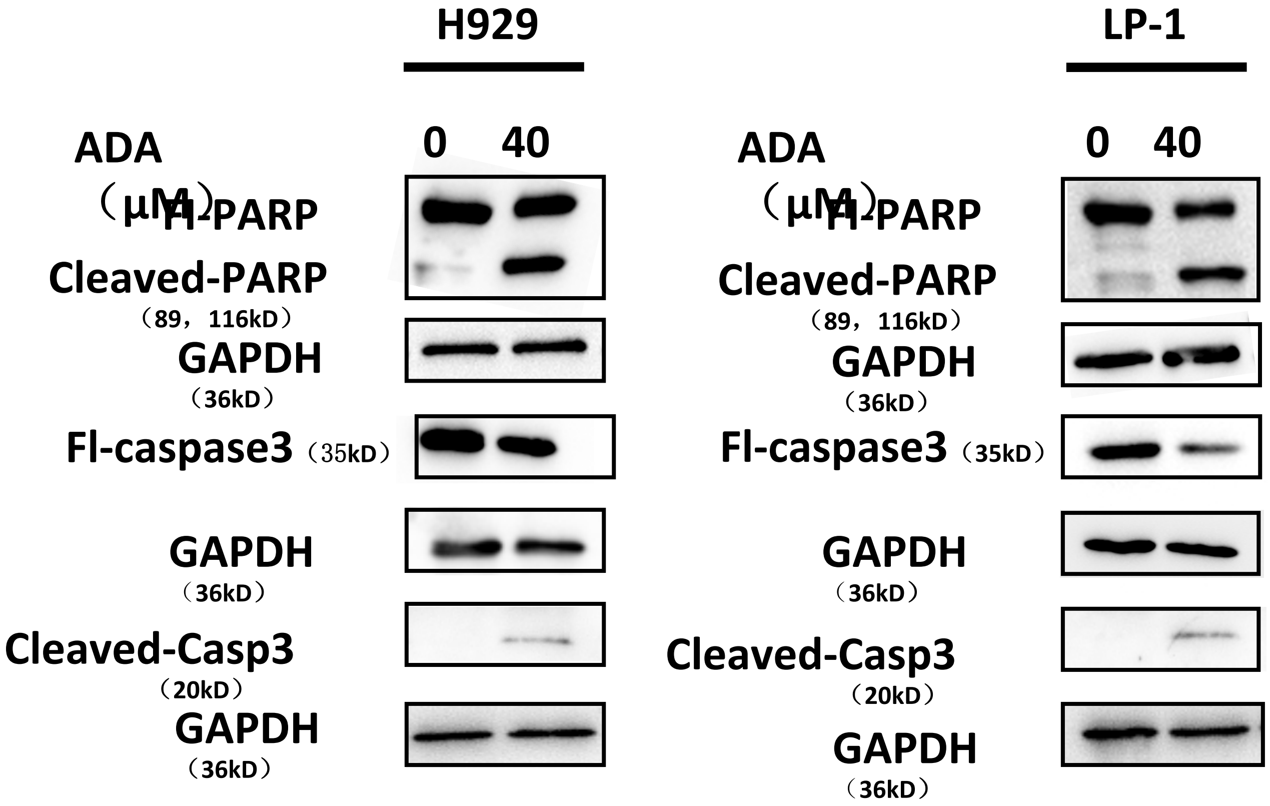
H929
LP-1
ADA（μM）
0
40
Fl-PARP
Cleaved-PARP
（89，116kD）
GAPDH
（36kD）
Fl-caspase3（35kD）
GAPDH
（36kD）
Cleaved-Casp3
（20kD）
GAPDH
（36kD）
ADA（μM）
0
40
Fl-PARP
Cleaved-PARP
（89，116kD）
GAPDH
（36kD）
Fl-caspase3（35kD）
GAPDH
（36kD）
Cleaved-Casp3
（20kD）
GAPDH
（36kD）

PARP

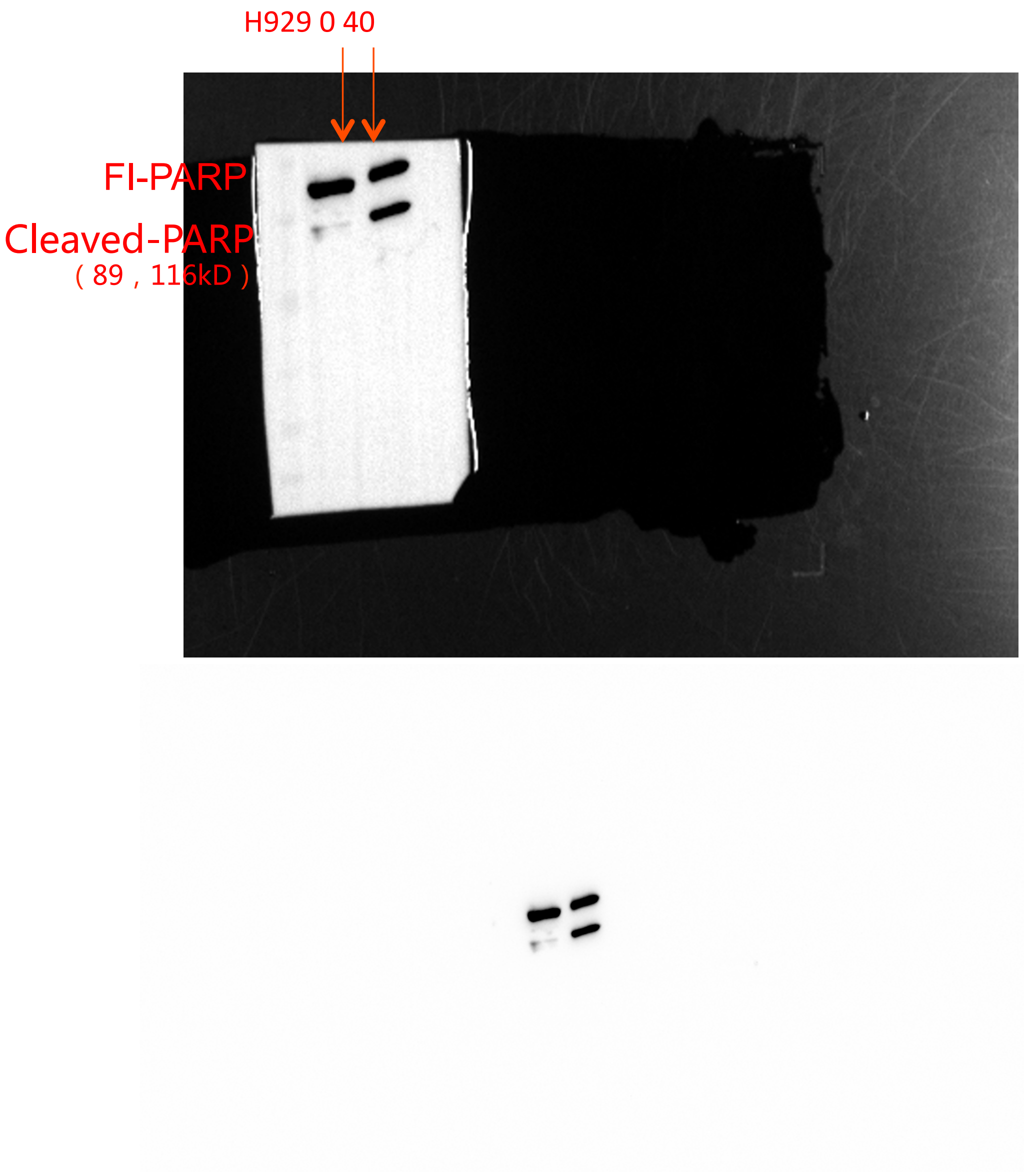
H929 0 40
Fl-PARP
Cleaved-PARP
(89 , 116kD)

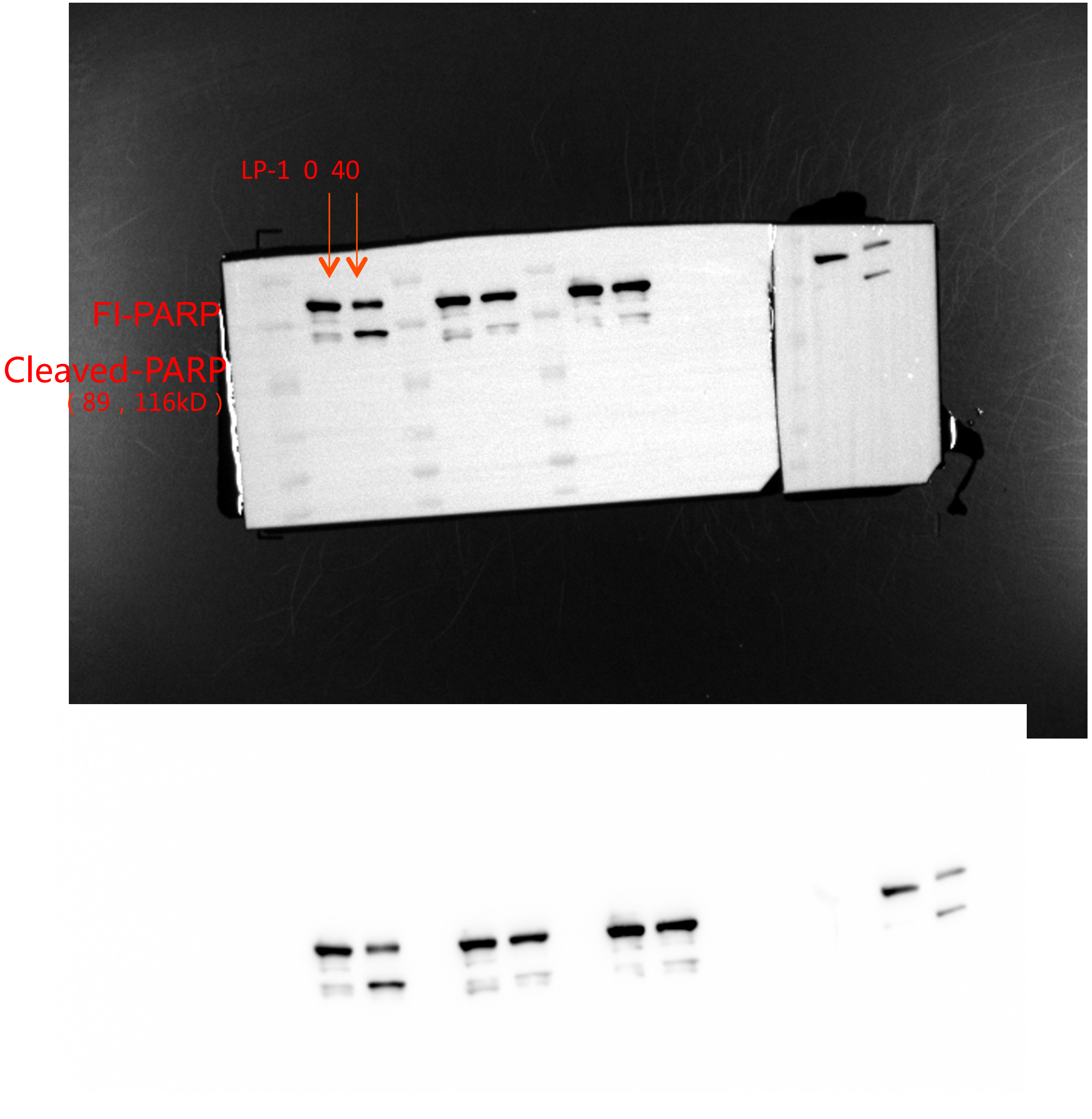
LP-1 0 40
Fl-PARP
Cleaved-PARP
(89 , 116kD)

GAPDH

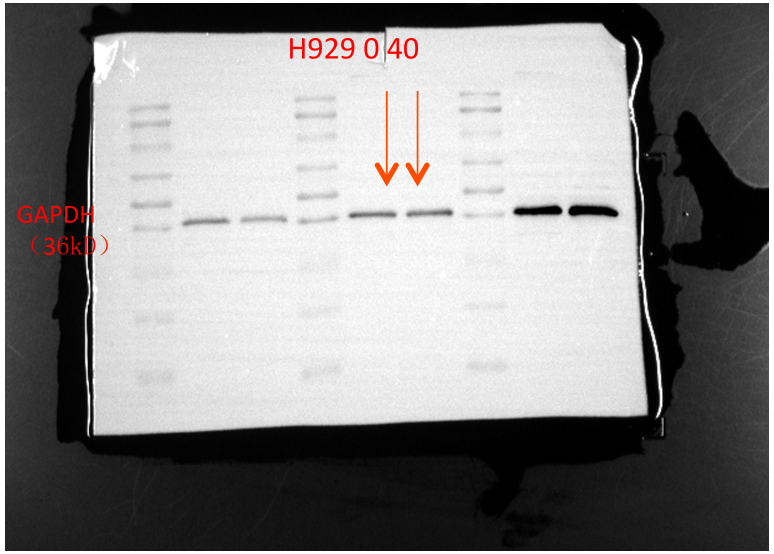

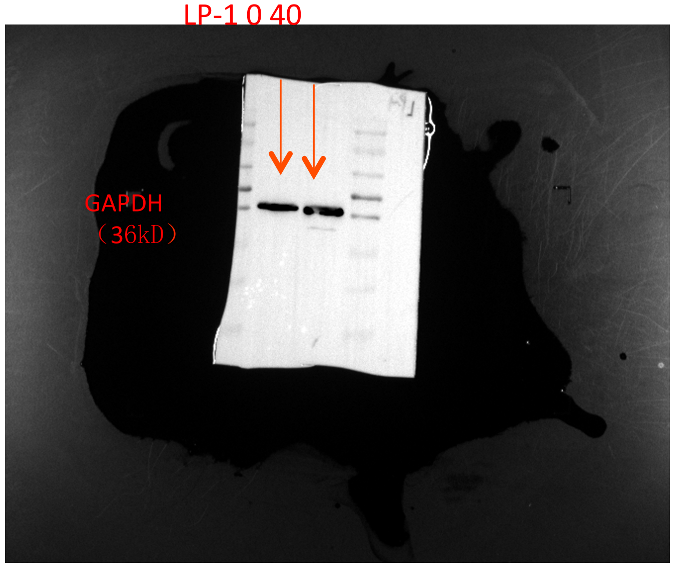

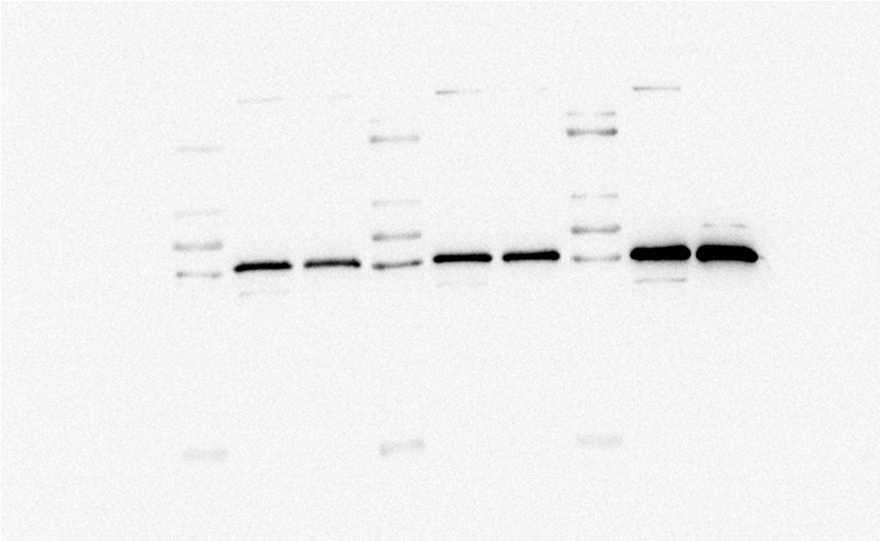

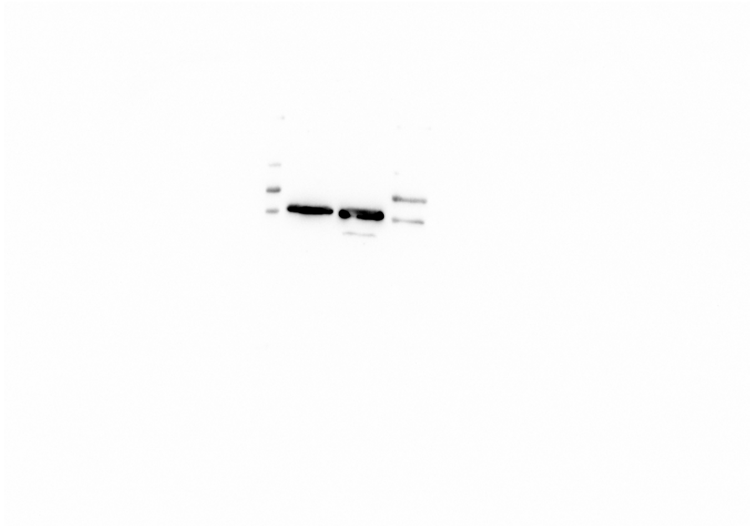

CASPASE 3

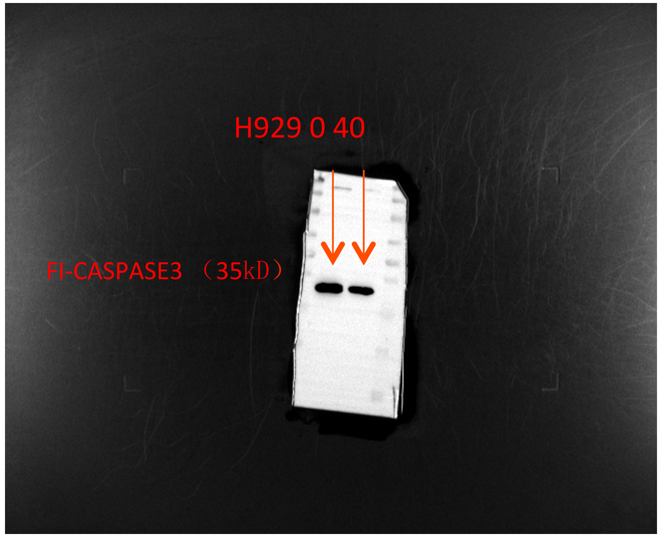

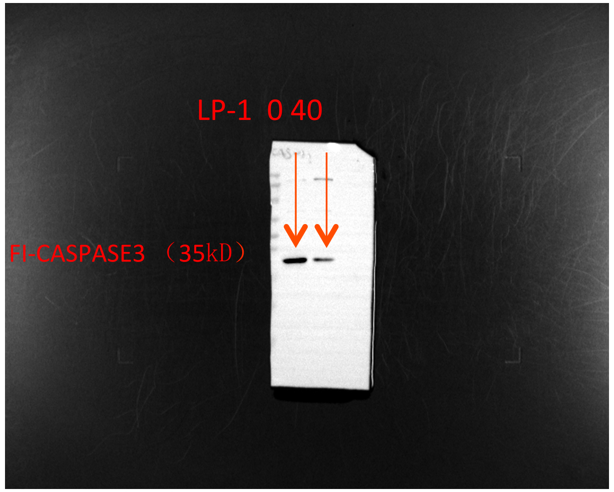

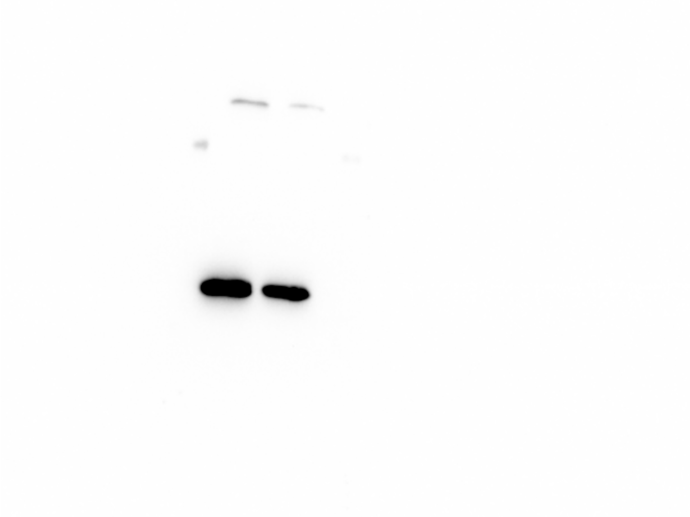

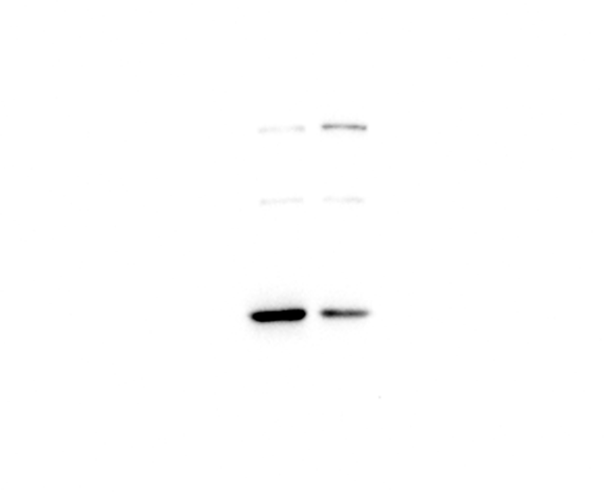

GAPDH

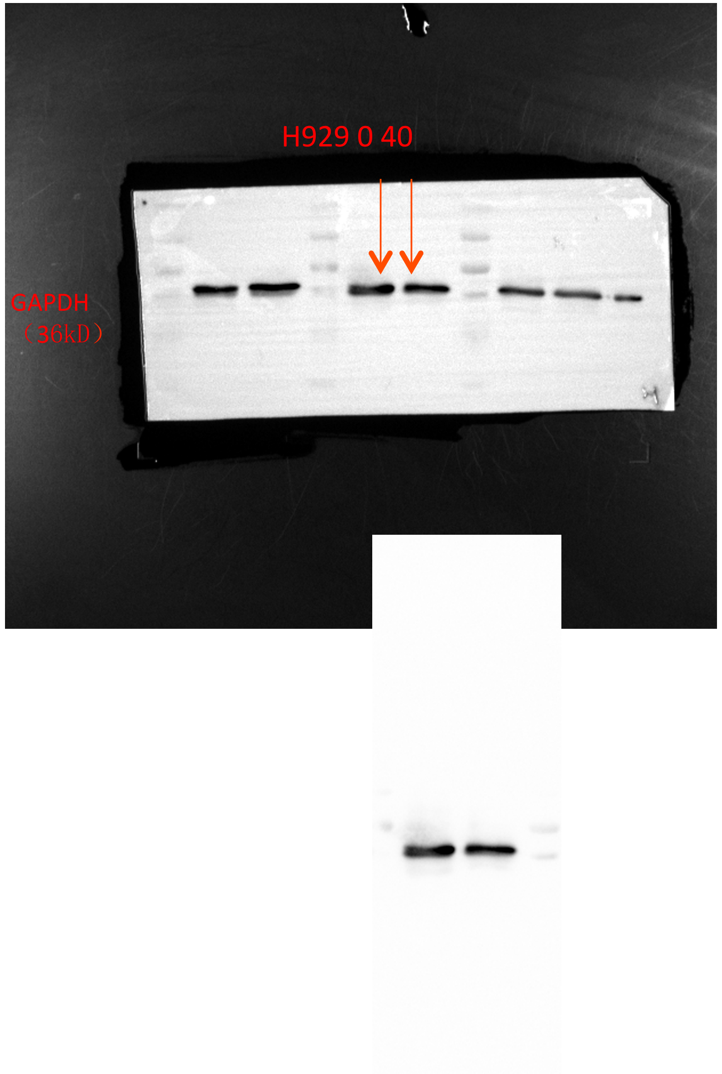

H929 GAPDH 10.10

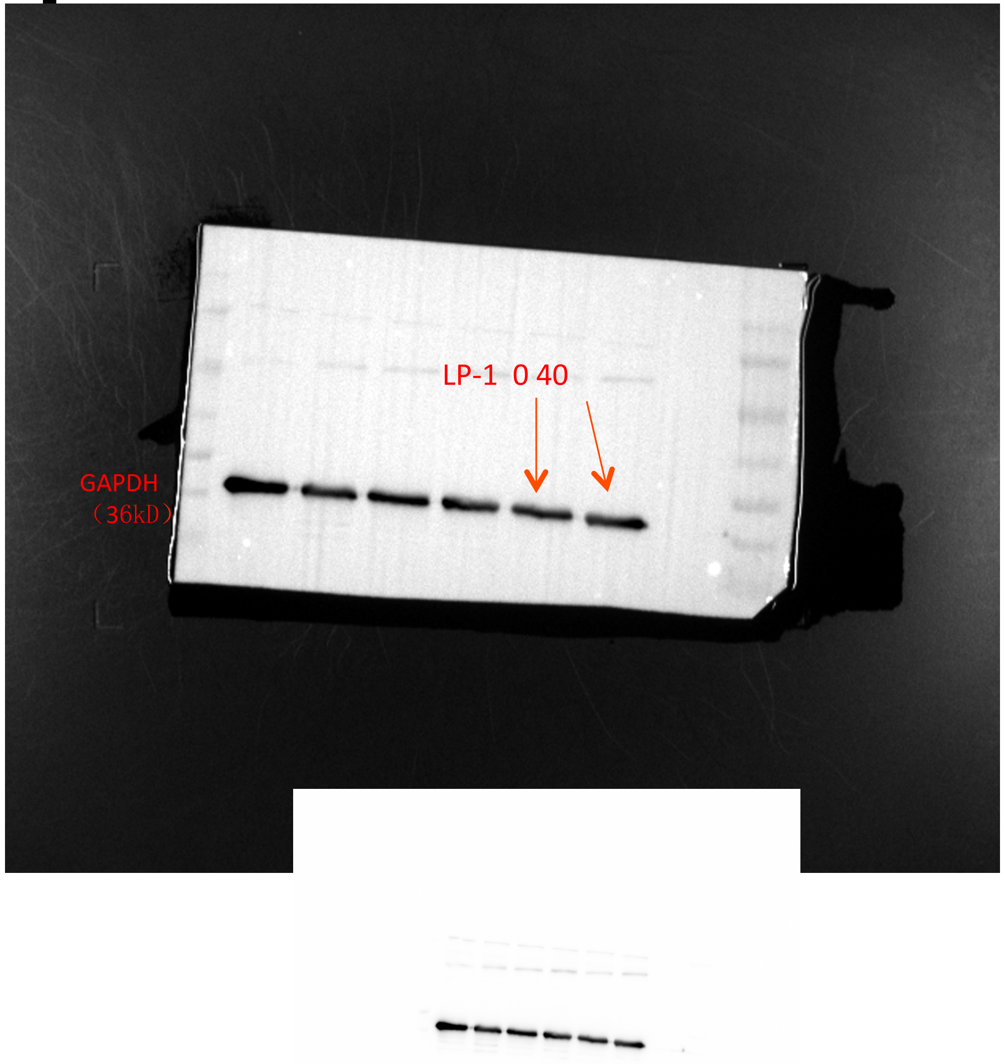

Cleaved-Casp3

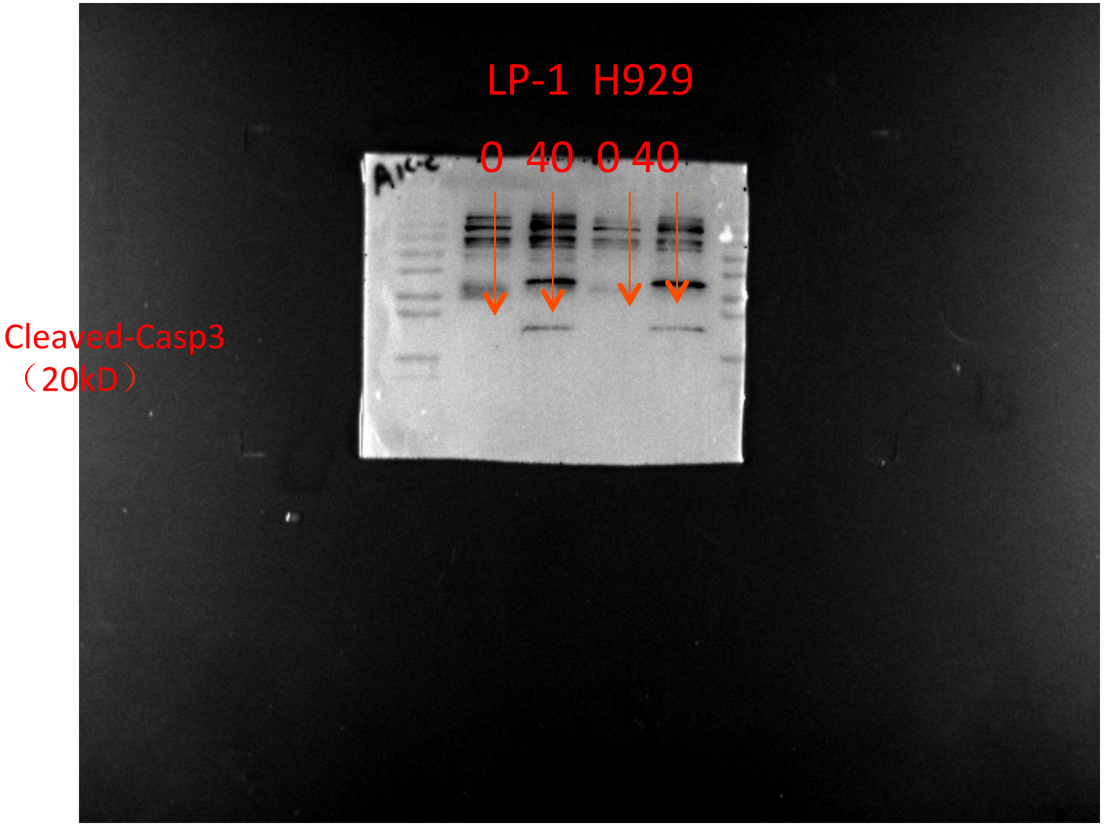

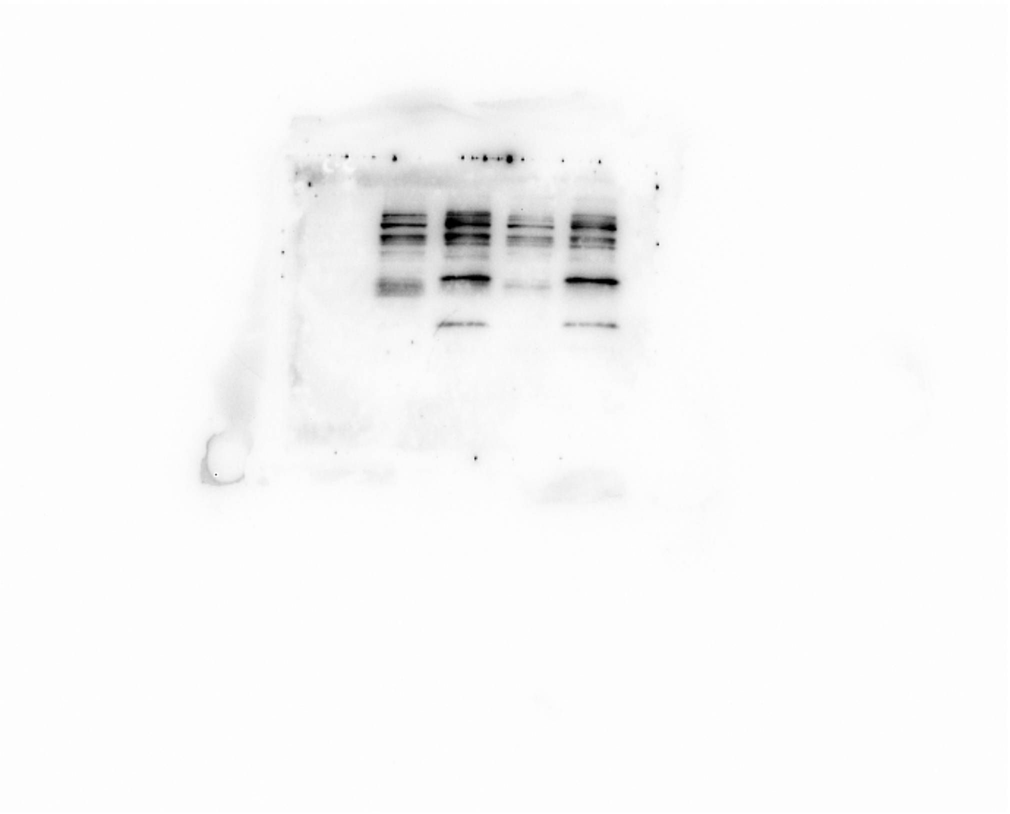

GAPDH

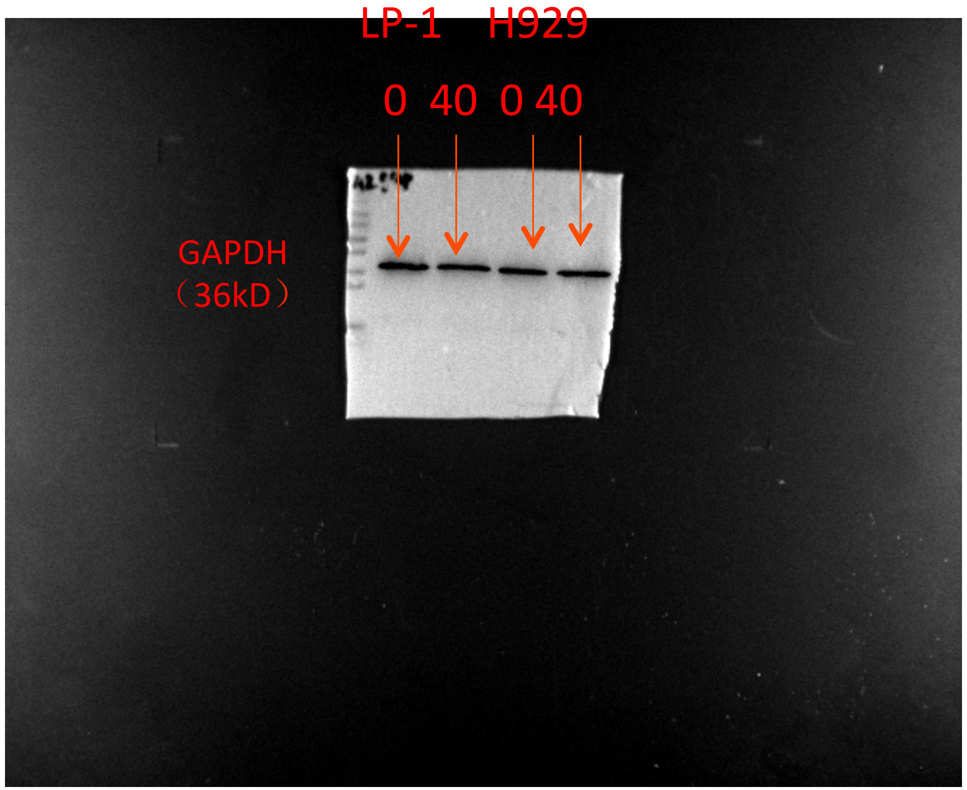

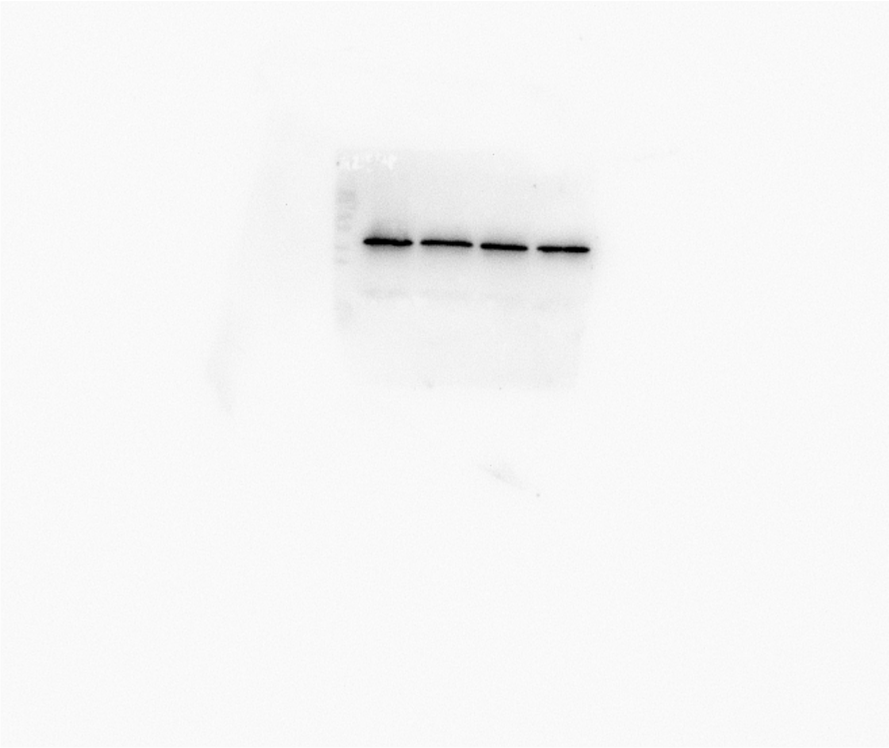

GAP
先lp-1 后h929

Figure 4D

BCL-2

H929 LP-1

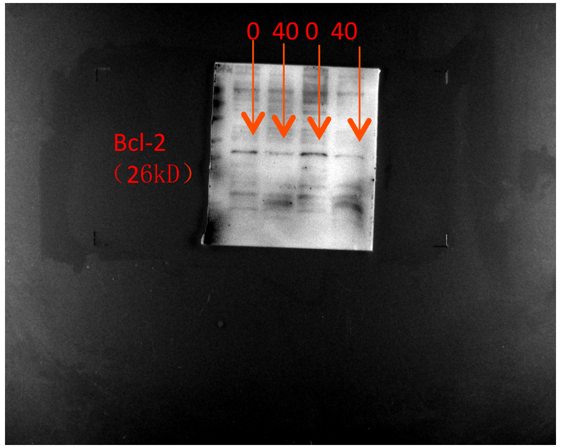

GAPDH

H929 LP-1

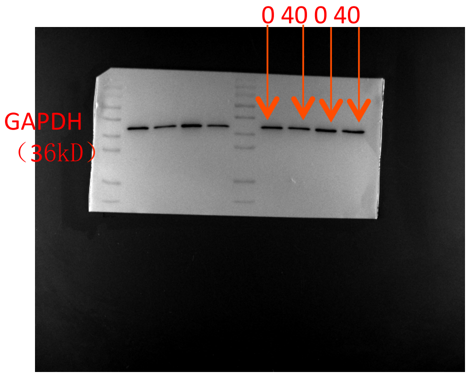

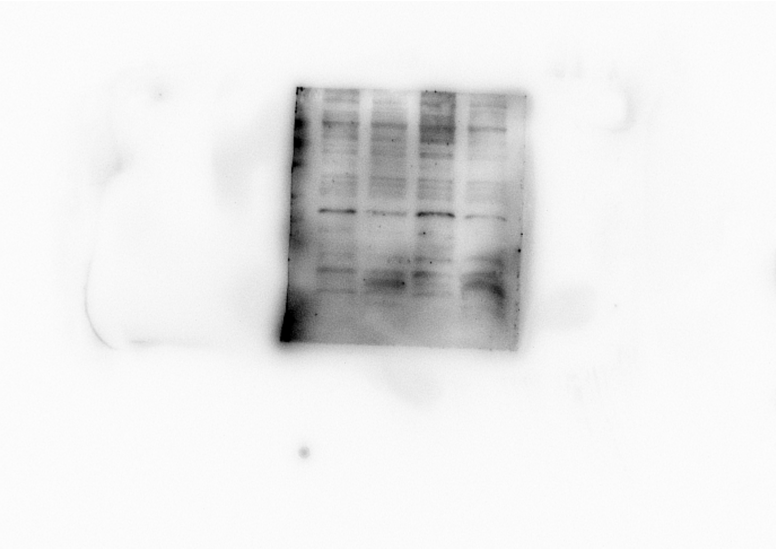

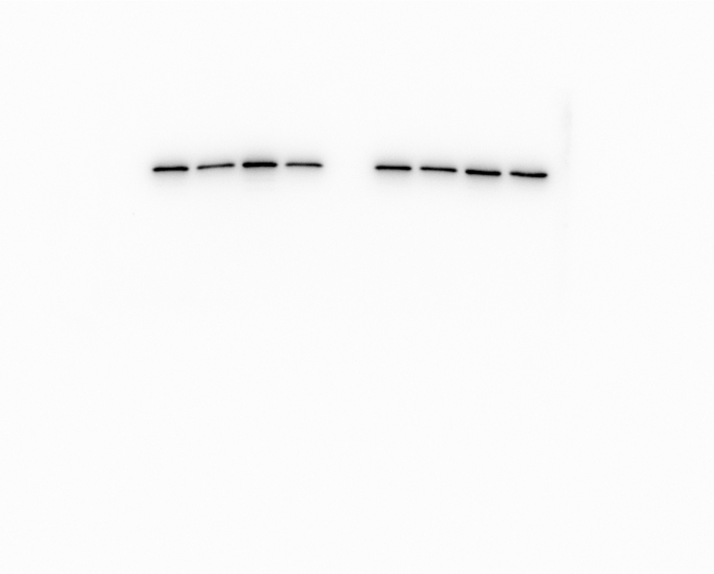

Figure 5D

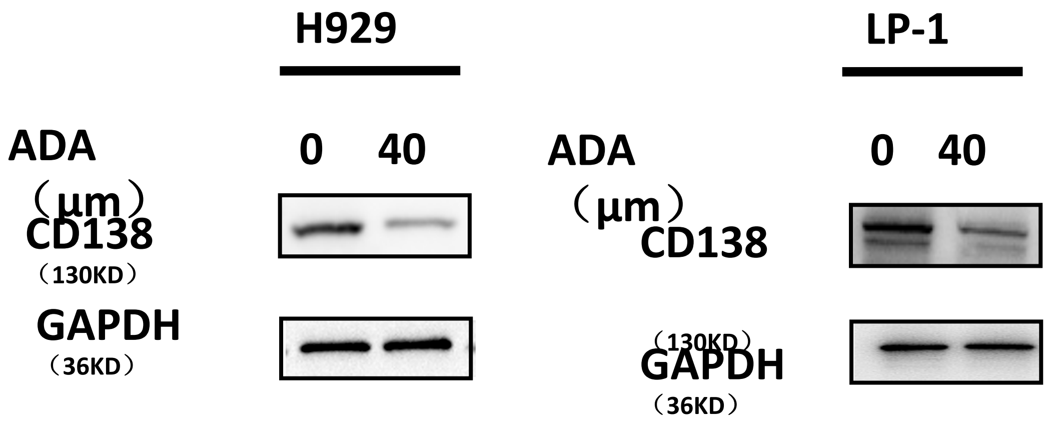

H929
ADA （μm）
0 40
CD138 （130KD）
GAPDH （36KD）
LP-1
ADA （μm）
0 40
CD138
（130KD）
GAPDH
（36KD）

CD138

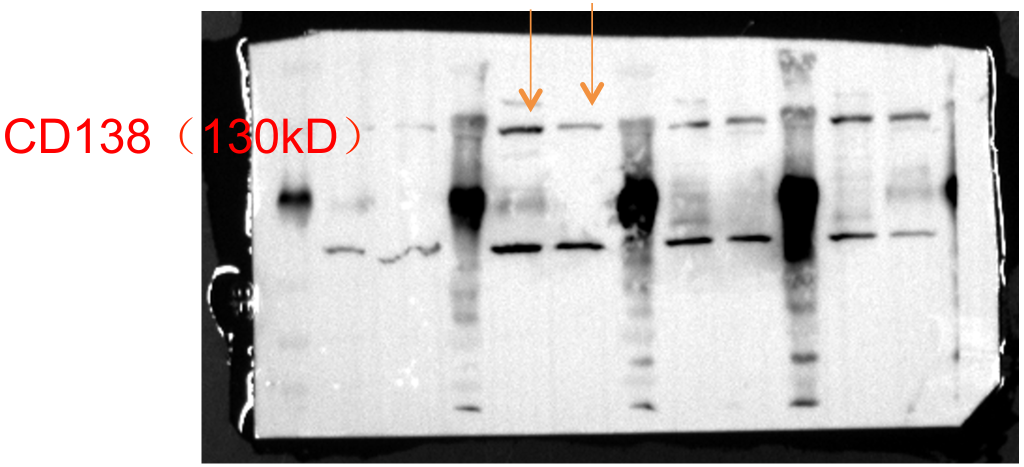

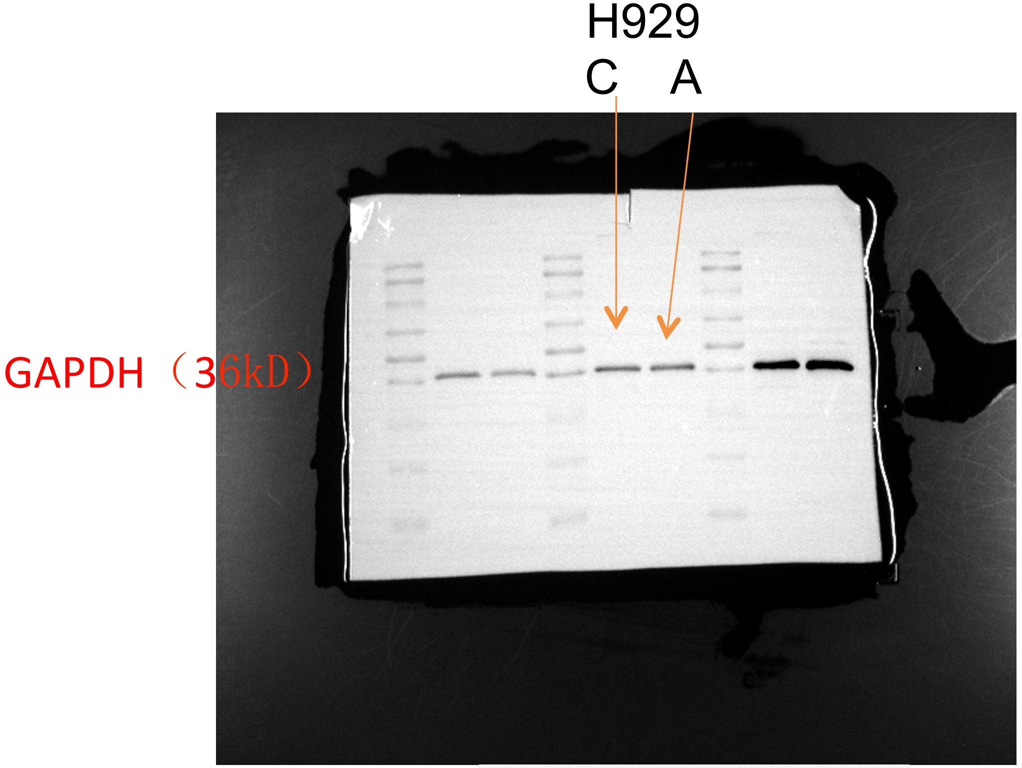

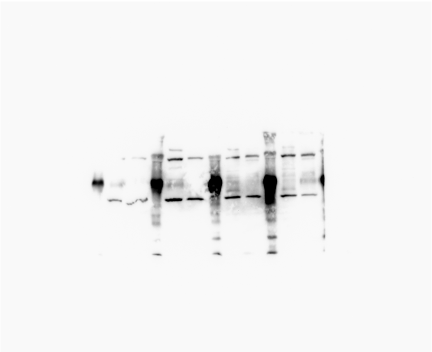

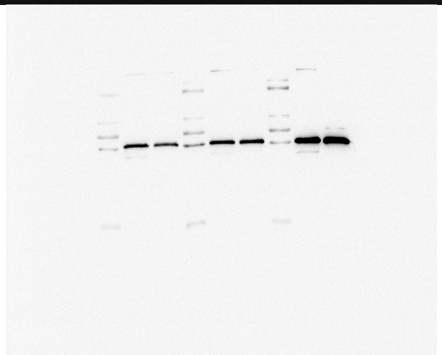

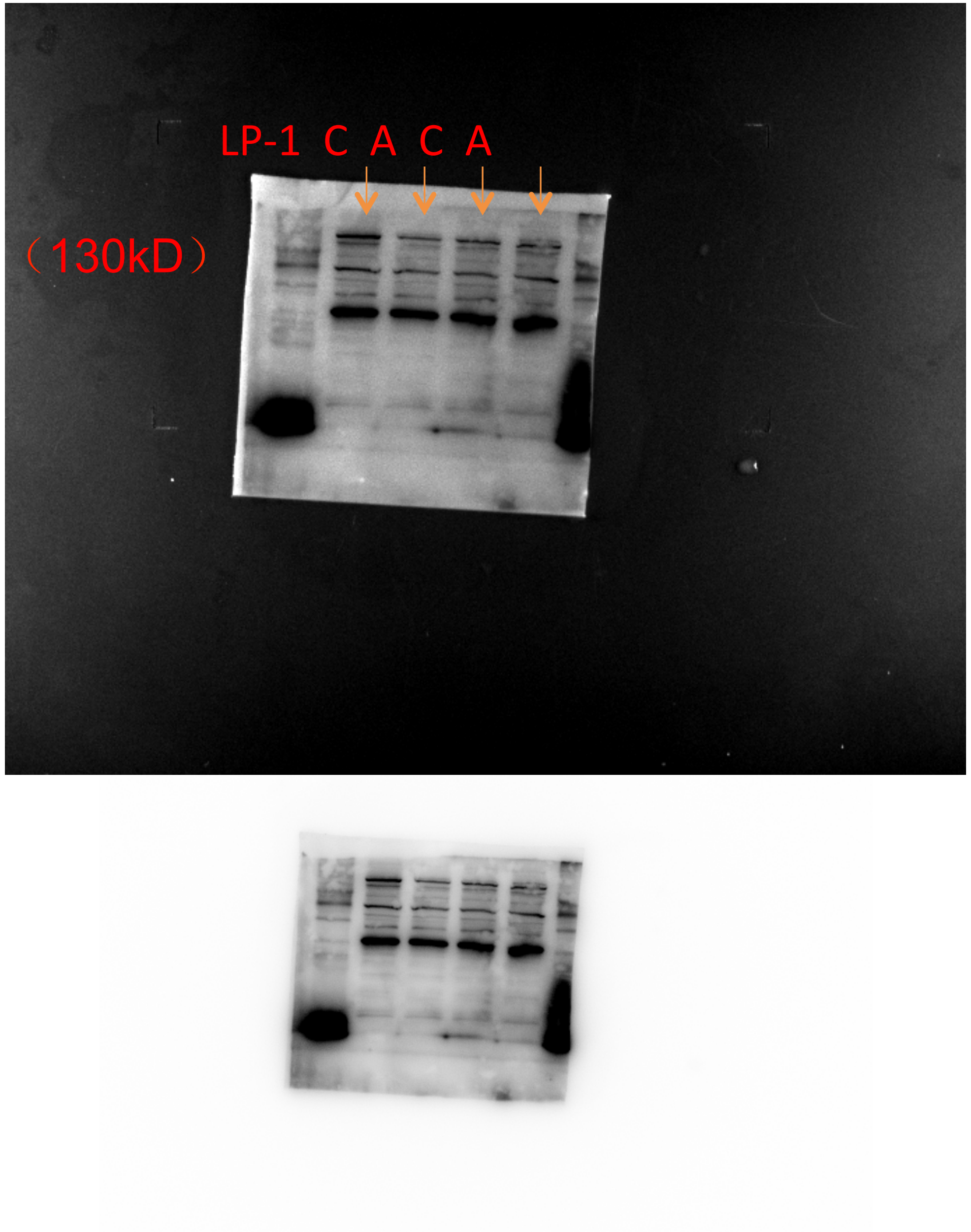
CD138（130kD）
LP-1 C A C A

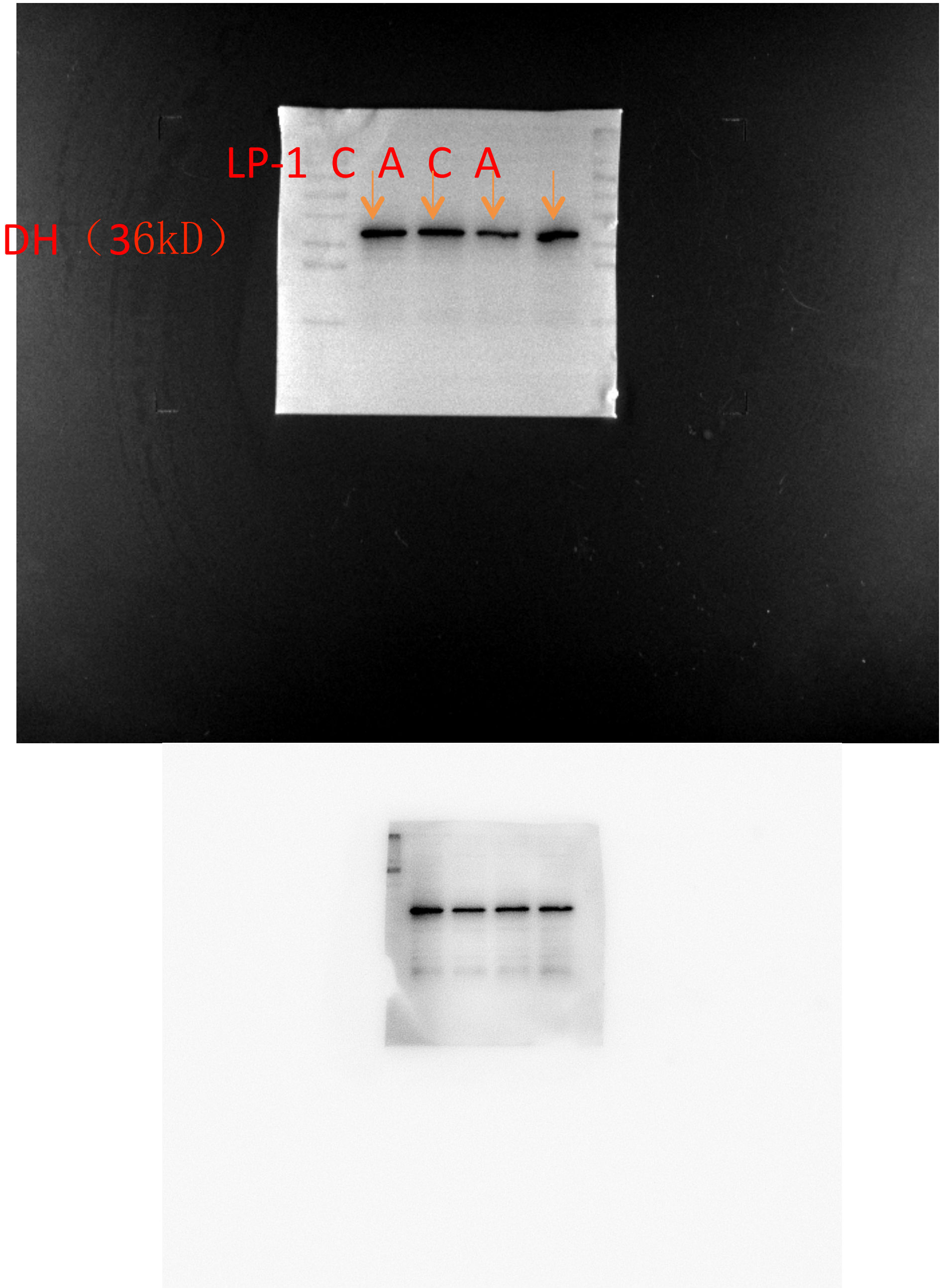
GAPDH（36kD）
LP-1 C A C A

Figure 6A

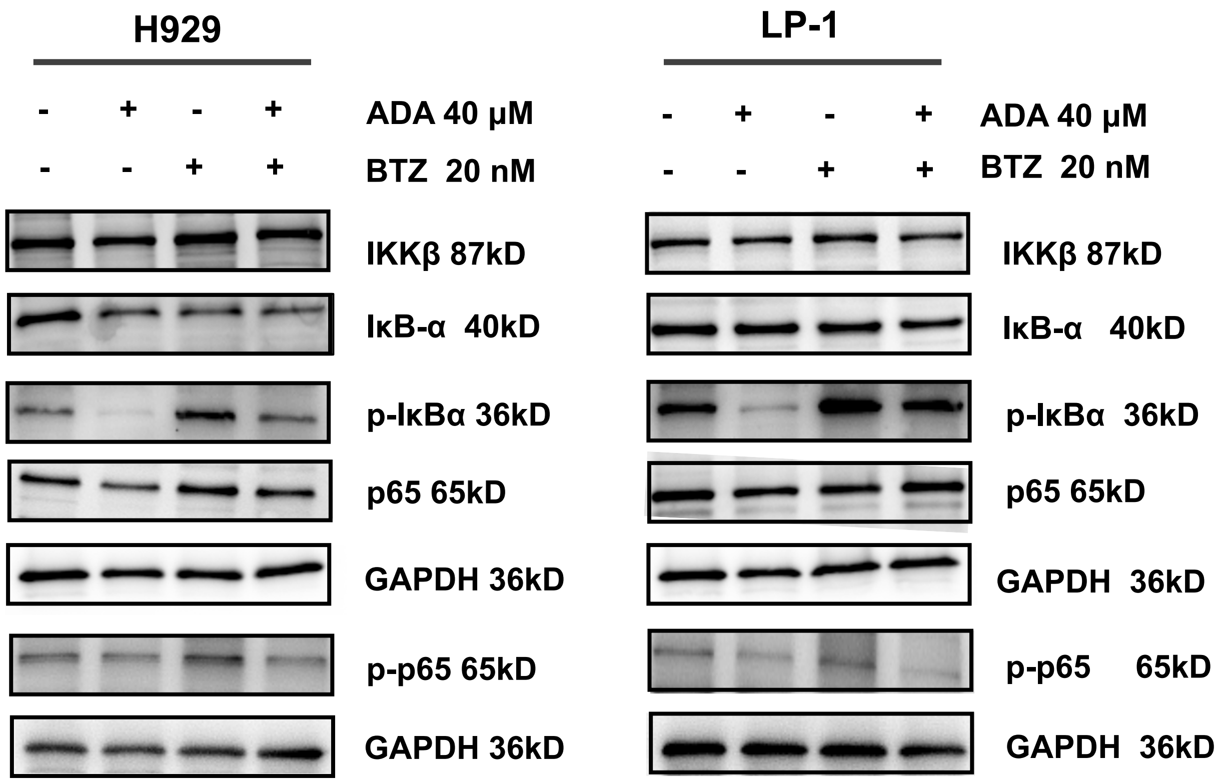
H929
LP-1
ADA 40 μM
BTZ 20 nM
IKKβ 87kD
IκB-α 40kD
p-IκBα 36kD
p65 65kD
GAPDH 36kD
p-p65 65kD
GAPDH 36kD

LP-1 H929

0 ADA BTZ ADA+BTZ 0 ADA BTZ ADA+BTZ

GAPDH 36kD

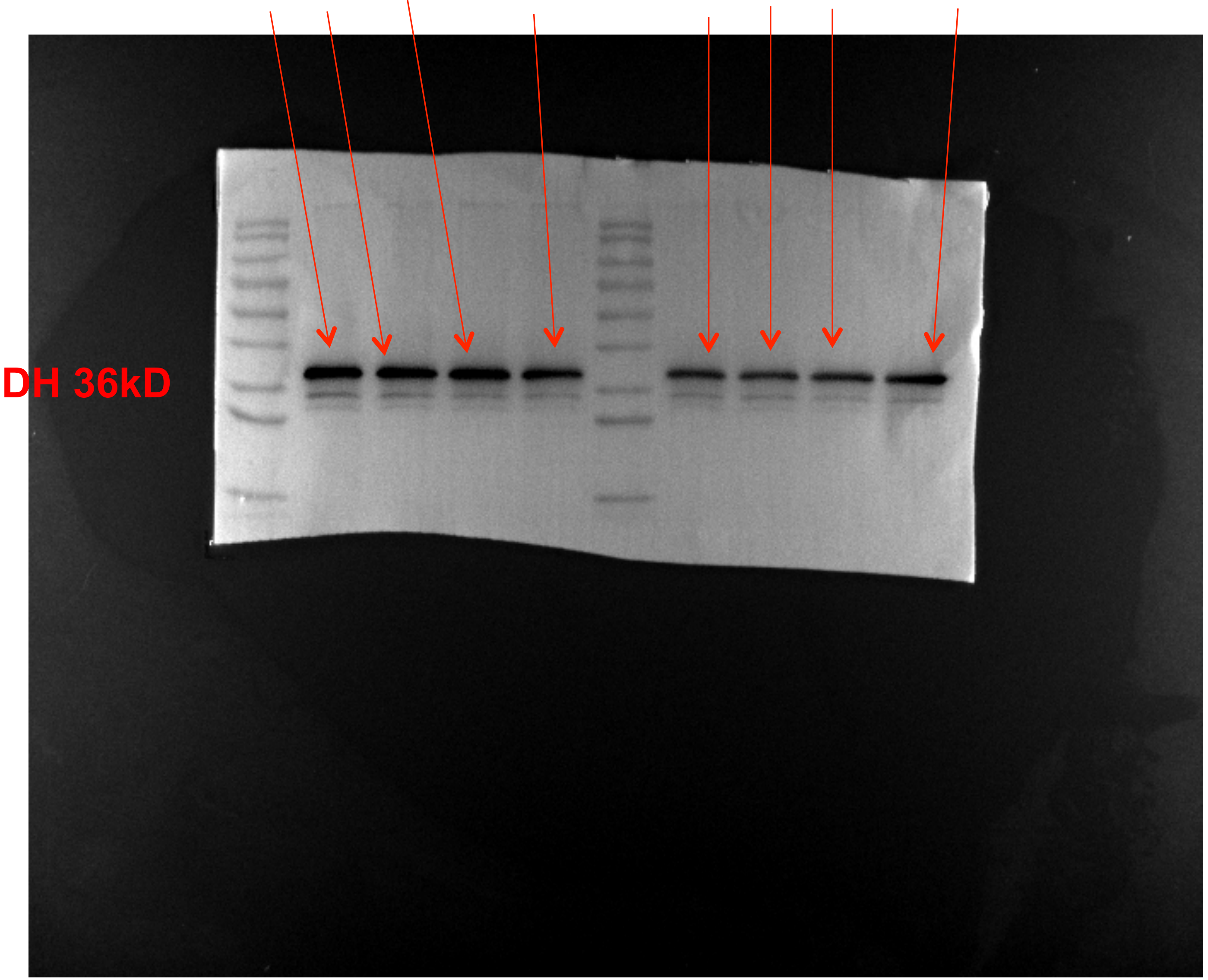

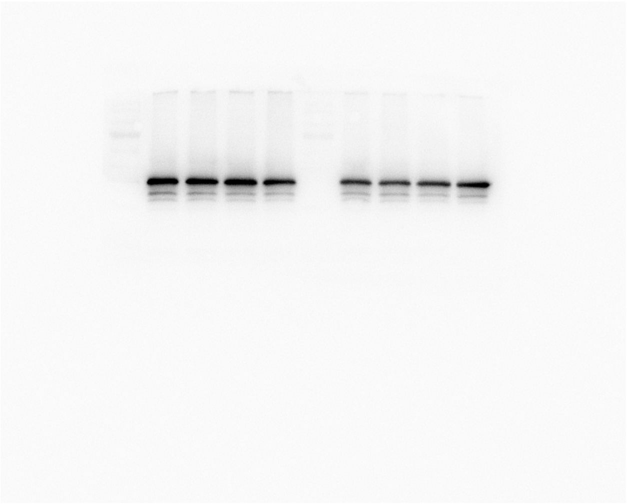

p-IkBα

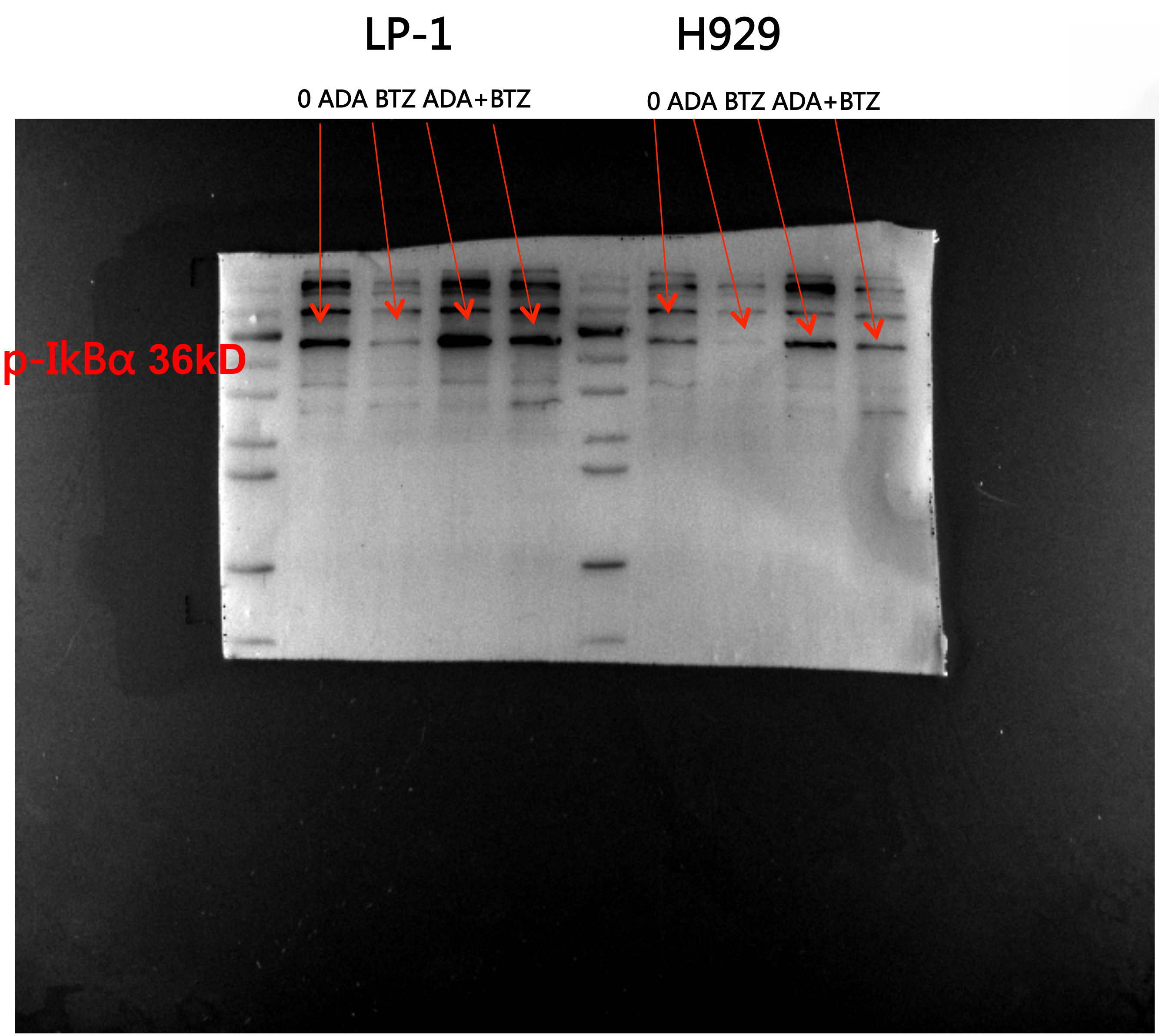

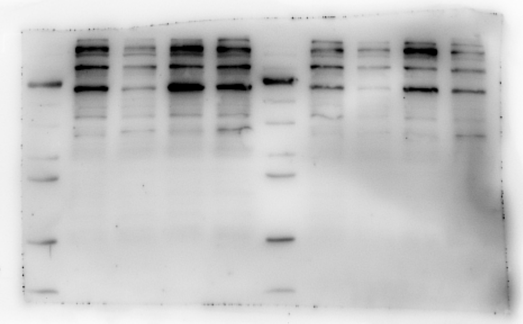

gap 2.14 lp-1 h929

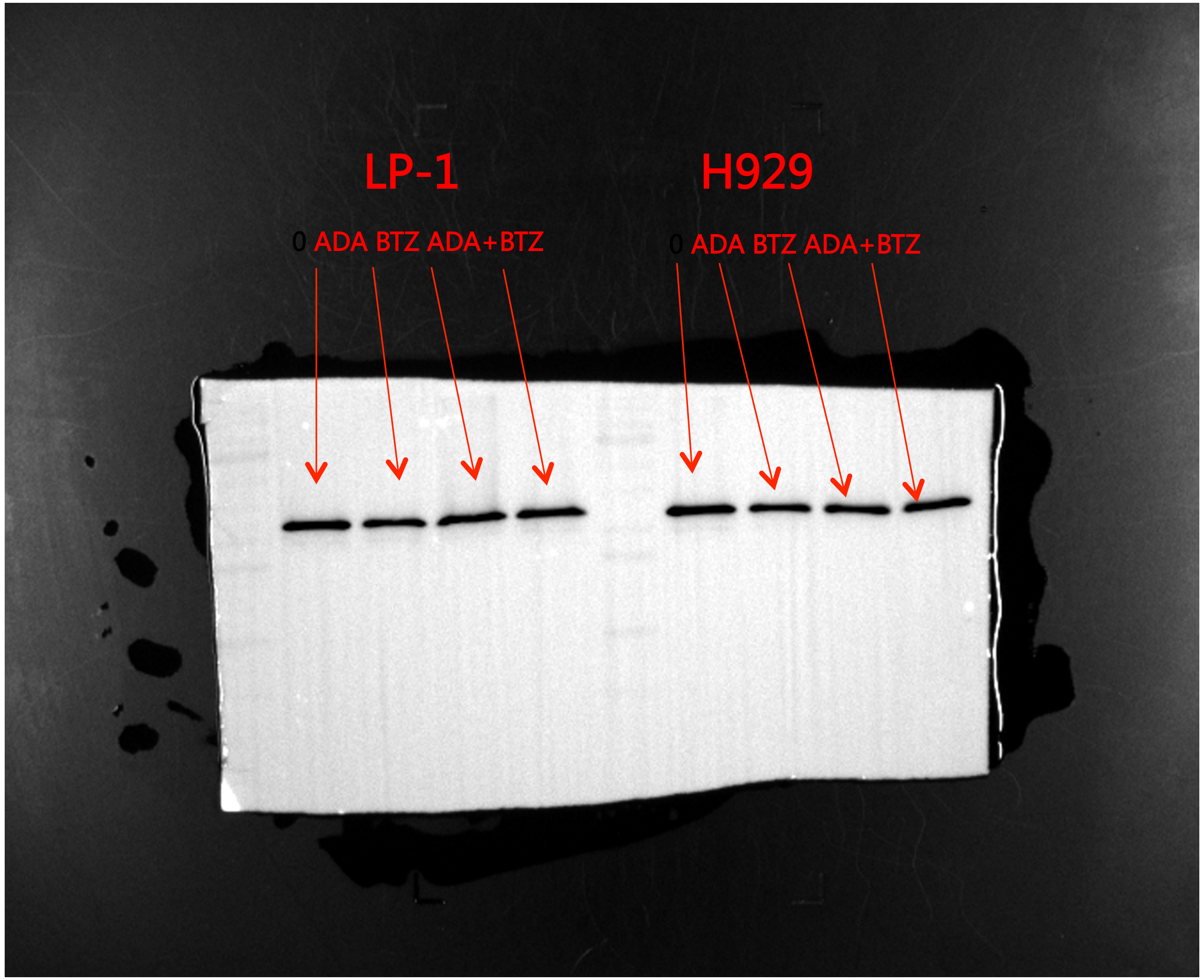

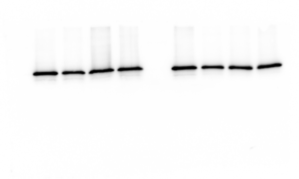

p65 2.16 H929

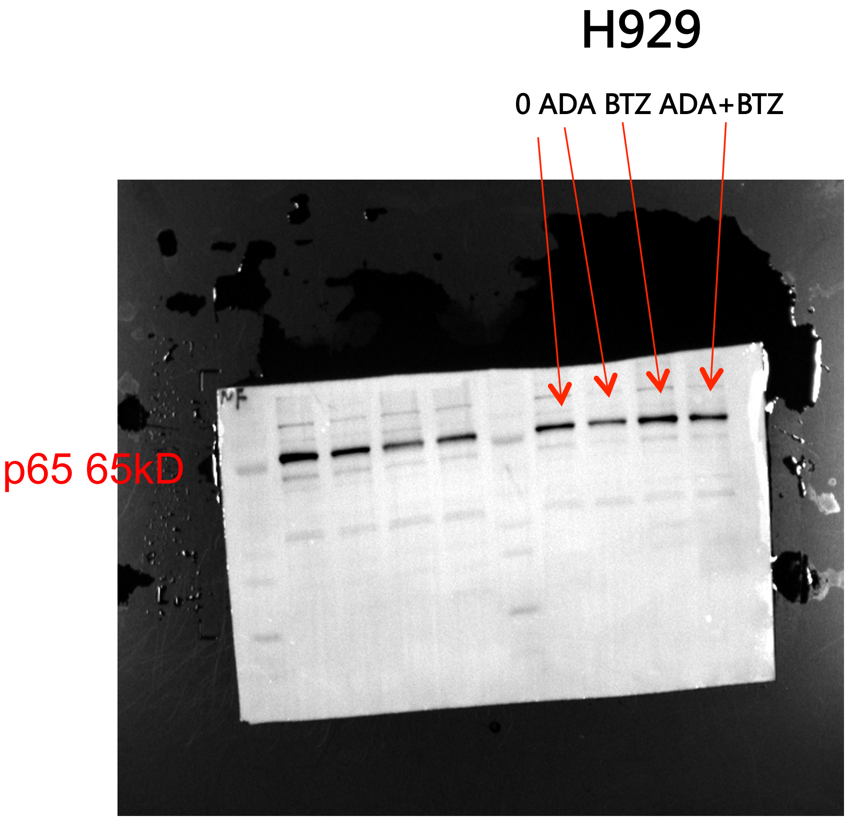

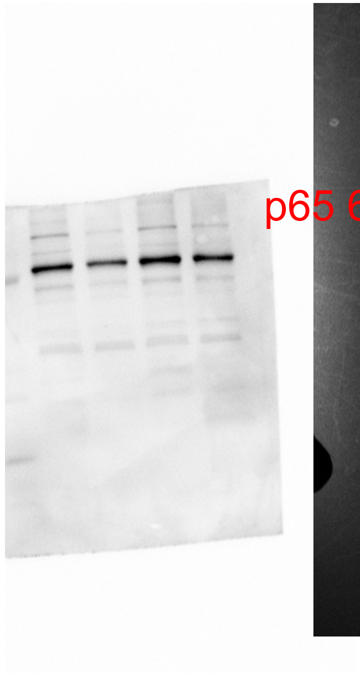

p65 2.16 LP-1

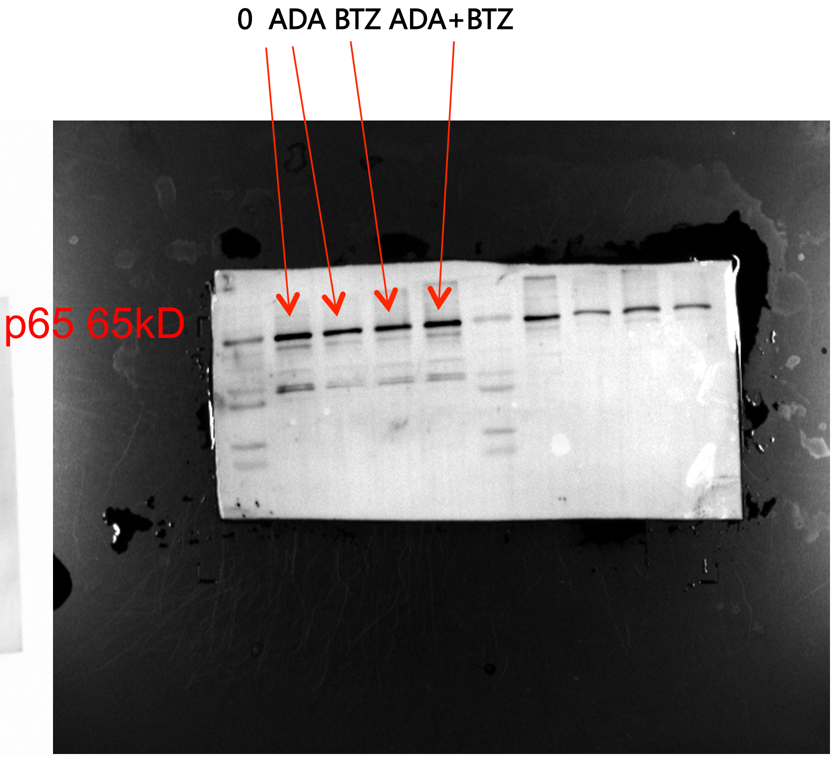

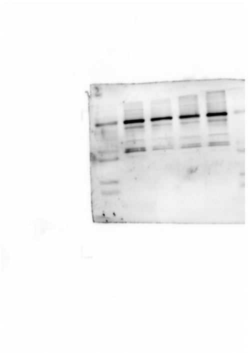

ikkb 2.14 LP-1 H929

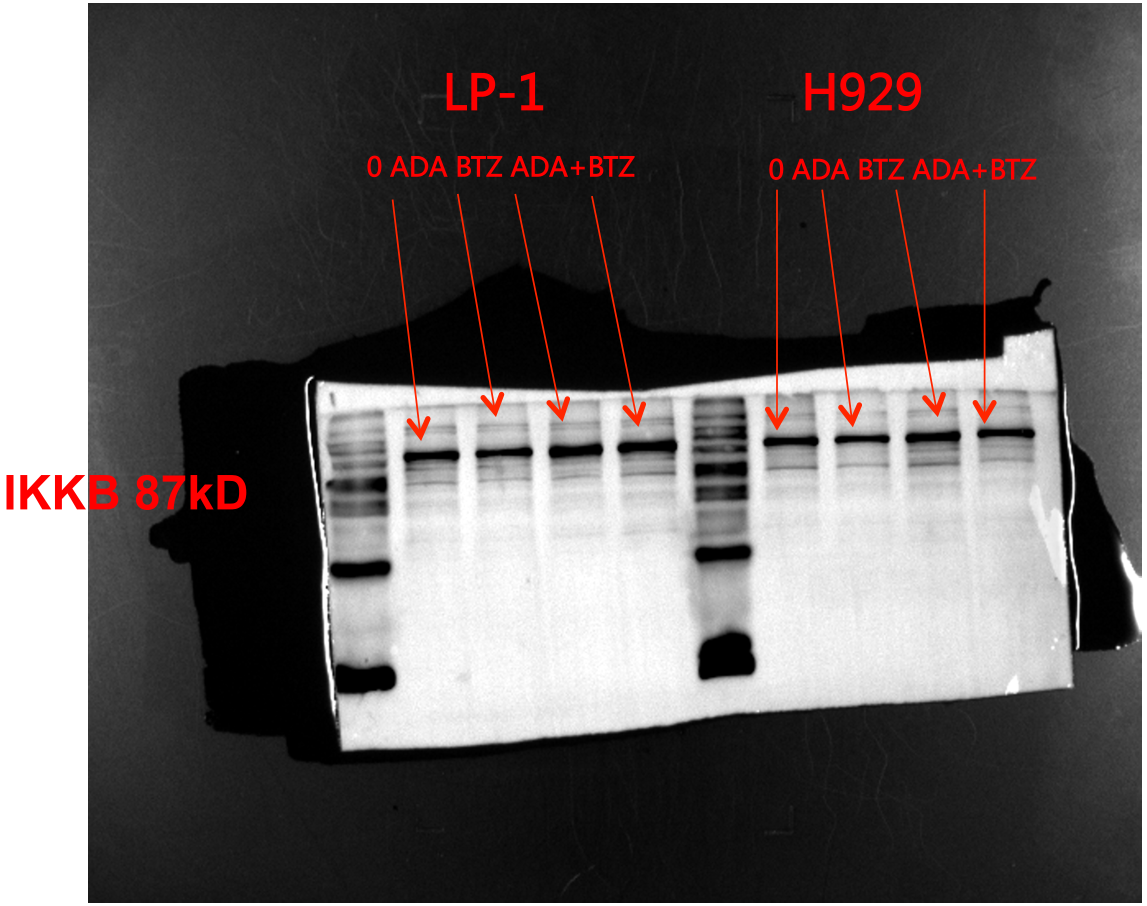

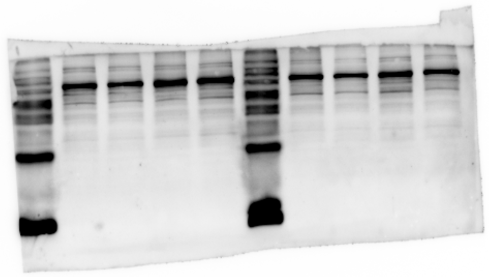

IκB-α 2.14 LP-1 H929

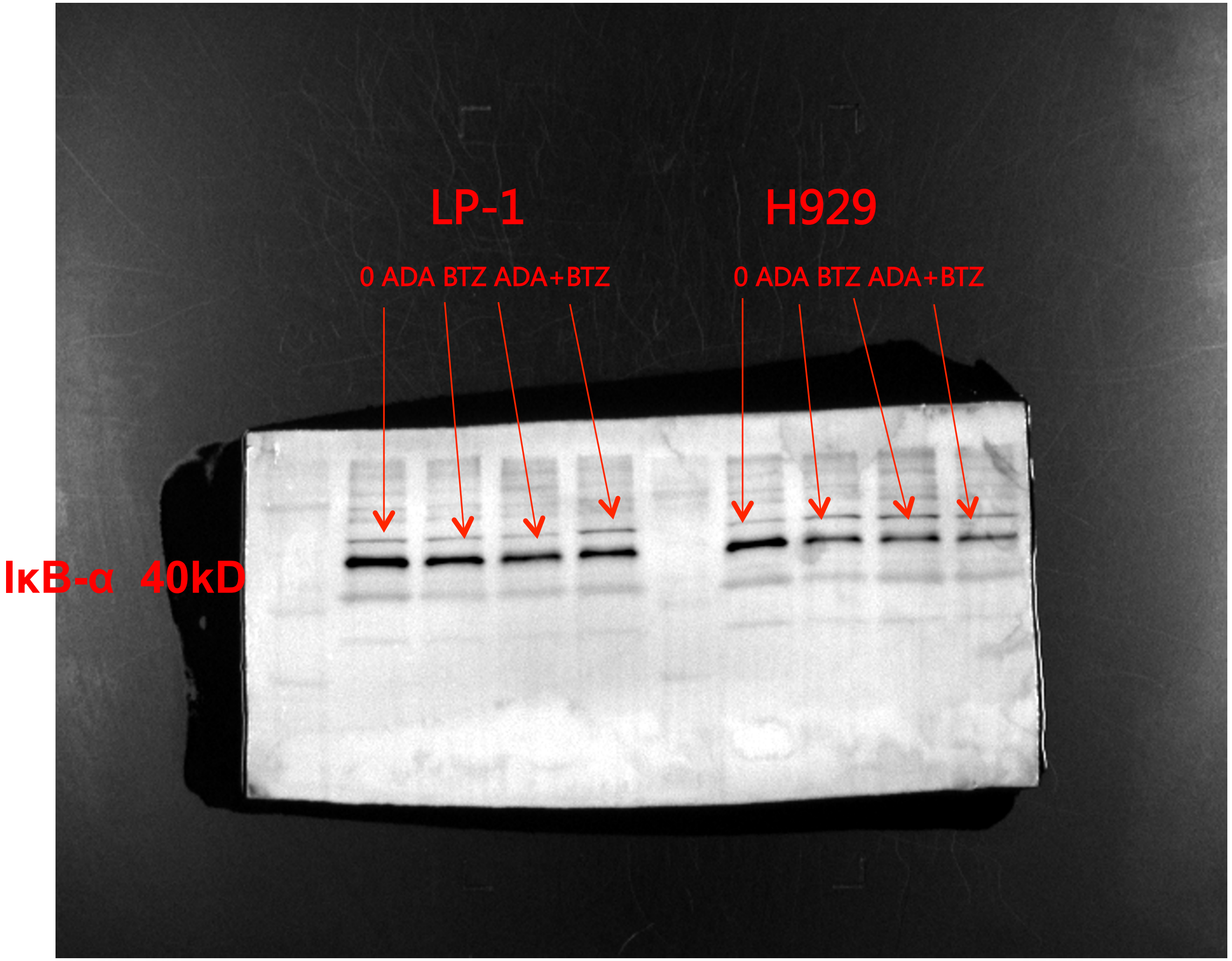

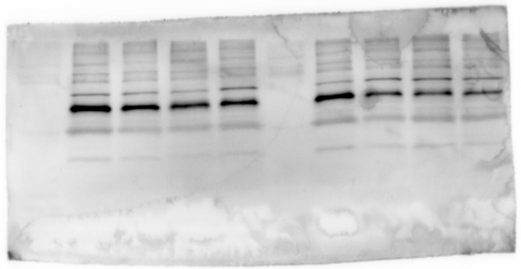

pp65 2.14 LP-1 H929

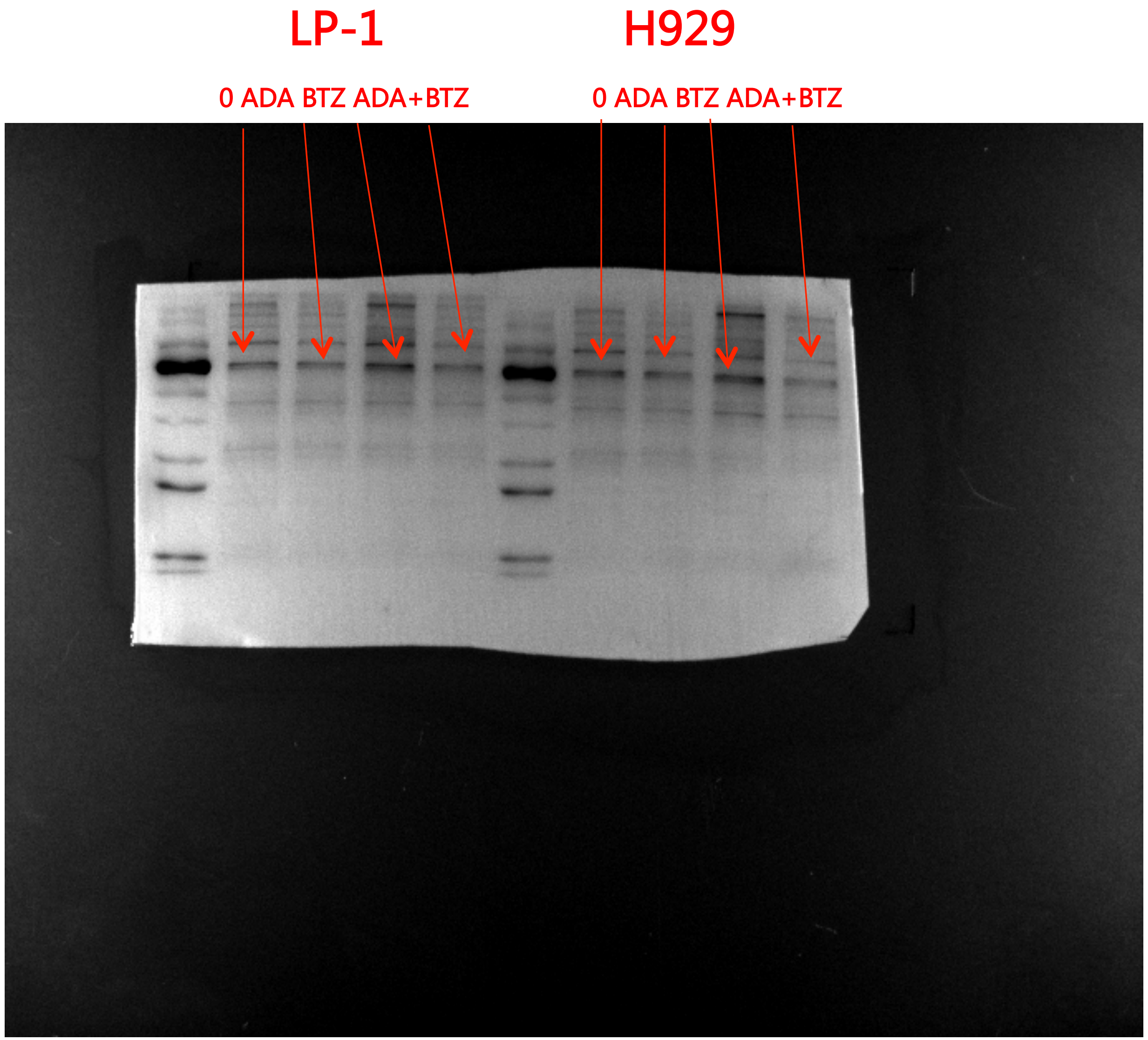

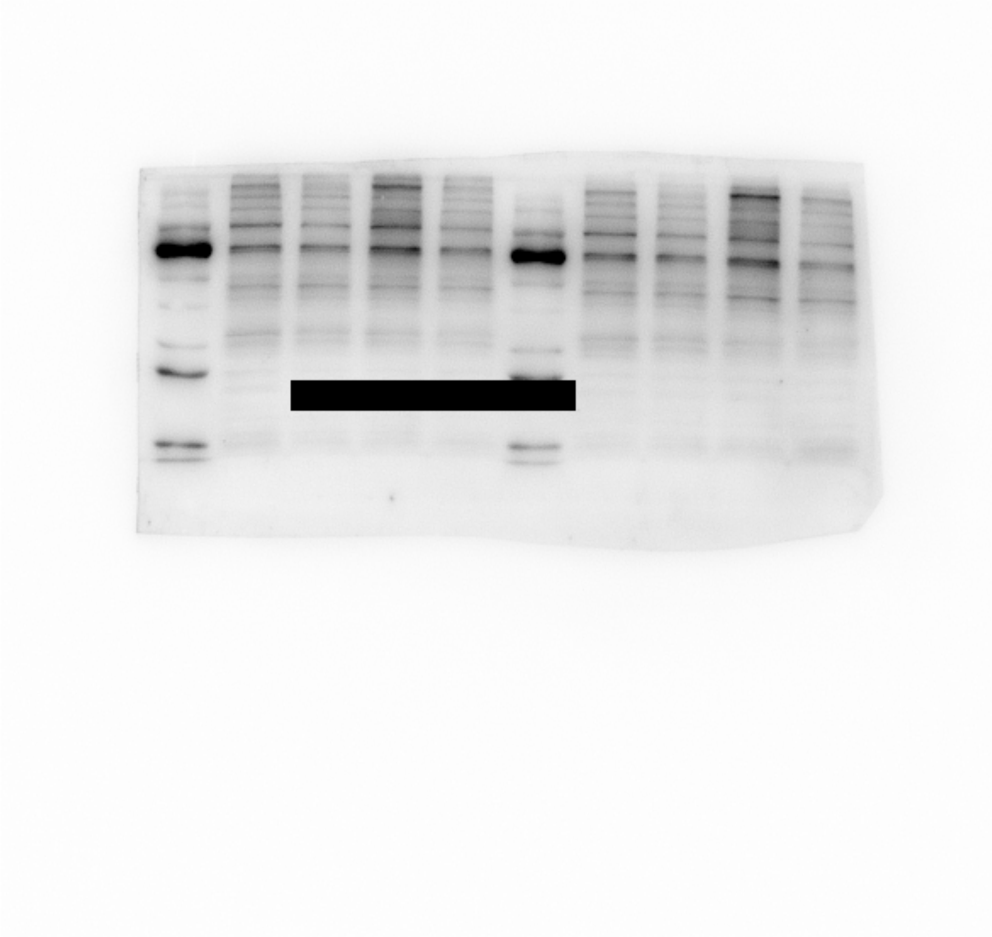

Supplement: Supplementary file 2 [file DataSheet1.PDF]
